# Active Ingredients and Mechanisms of Change in Motivational Interviewing for Medication Adherence. A Mixed Methods Study of Patient-Therapist Interaction in Patients With Schizophrenia

**DOI:** 10.3389/fpsyt.2020.00078

**Published:** 2020-03-24

**Authors:** Jos Dobber, Corine Latour, Berno van Meijel, Gerben ter Riet, Emile Barkhof, Ron Peters, Wilma Scholte op Reimer, Lieuwe de Haan

**Affiliations:** ^1^ACHIEVE Centre of Applied Research, Faculty of Health, Amsterdam University of Applied Sciences, Amsterdam, Netherlands; ^2^Research Group Mental Health Nursing, Inholland University of Applied Sciences, Amsterdam, Netherlands; ^3^Department of Psychiatry, Amsterdam UMC (VUmc), Amsterdam Public Health Research Institute, Amsterdam, Netherlands; ^4^Parnassia Academy, Parnassia Psychiatric Institute, The Hague, Netherlands; ^5^Department of General Practice, Amsterdam UMC, University of Amsterdam, Amsterdam, Netherlands; ^6^GGZ Rivierduinen, Leiden, Netherlands; ^7^Department of Cardiology, Amsterdam UMC, University of Amsterdam, Amsterdam, Netherlands; ^8^Department of Psychiatry, Amsterdam UMC, University of Amsterdam, Amsterdam, Netherlands

**Keywords:** motivational interviewing, schizophrenia, medication adherence, active ingredients, mechanisms of change

## Abstract

**Background:**

Trials studying Motivational Interviewing (MI) to improve medication adherence in patients with schizophrenia showed mixed results. Moreover, it is unknown which active MI-ingredients are associated with mechanisms of change in patients with schizophrenia. To enhance the effect of MI for patients with schizophrenia, we studied MI's active ingredients and its working mechanisms.

**Methods:**

First, based on MI literature, we developed a model of potential active ingredients and mechanisms of change of MI in patients with schizophrenia. We used this model in a qualitative multiple case study to analyze the application of the active ingredients and the occurrence of mechanisms of change. We studied the cases of fourteen patients with schizophrenia who participated in a study on the effect of MI on medication adherence. Second, we used the Generalized Sequential Querier (GSEQ 5.1) to perform a sequential analysis of the MI-conversations aiming to assess the transitional probabilities between therapist use of MI-techniques and subsequent patient reactions in terms of change talk and sustain talk.

**Results:**

We found the therapist factor “a trusting relationship and empathy” important to enable sufficient depth in the conversation to allow for the opportunity of triggering mechanisms of change. The most important conversational techniques we observed that shape the hypothesized active ingredients are reflections and questions addressing medication adherent behavior or intentions, which approximately 70% of the time was followed by “patient change talk”. Surprisingly, sequential MI-consistent therapist behavior like “affirmation” and “emphasizing control” was only about 6% of the time followed by patient change talk. If the active ingredients were embedded in more comprehensive MI-strategies they had more impact on the mechanisms of change.

**Conclusions:**

Mechanisms of change mostly occurred after an interaction of active ingredients contributed by both therapist and patient. Our model of active ingredients and mechanisms of change enabled us to see “MI at work” in the MI-sessions under study, and this model may help practitioners to shape their MI-strategies to a potentially more effective MI.

## Introduction

Antipsychotic drug treatment is an effective intervention in patients with schizophrenia (1). However, non-adherence is a problem in approximately 42%–74% of the patients (2, 3). Motivational Interviewing (MI) may be an intervention to stimulate motivation for long-term medication adherence. However, studies on the use of MI to promote medication adherence in schizophrenia show mixed results (4–7), in contrast to the more consistent effects of MI on behavior change in many other disorders (8–11). These discrepancies may partially be explained by differences in MI-strategy, in particular by the application of active ingredients, leading to success or failure in subsequent activation of mechanisms of change in the patients (12). Furthermore, these ingredients and mechanisms of successful MI may be different for patients with schizophrenia (13).

MI is “a collaborative conversation style for strengthening a person's own motivation and commitment to change” [(14), p.29], it addresses the common problem of ambivalence about change. Patients may feel ambivalent about medication adherence, e.g., knowing it may help to prevent psychotic relapse and readmission, and at the same time experiencing burdensome side effects, such as sedation or weight gain. In MI, the therapist deliberately influences the patient's motivation for change, through eliciting change talk (pro change) and softening sustain talk (counter change). The therapist adopts an empathetic attitude, thus communicating the partnership with the patient. The intervention includes four overlapping processes: engaging (relation building), focusing (finding the patient's change goals), evoking (eliciting change talk: the patient's own motives for change), and planning (supporting the patient to create a small concrete plan to move on to actual change). (14).

Nock (15) described three classes of factors involved in psychological interventions to influence subsequent behavior change: clinician factors, client factors, and mechanisms of change (see **Table 1**). The clinician and client factors of interest are those that form the active ingredients of MI. In literature on MI-theory (12, 14, 16–18) and in research (19–23) there are several hypothesized active ingredients of MI in general, such as the clinician factor “discussing ambivalence” (17, 18), and the client factor “experiencing discrepancy” (19, 23). There are also some hypothesized mechanisms of change of MI, e.g. “arguing oneself into change” (14, 18). If we would know which active ingredients and which mechanisms of change determine the success of MI in patients with schizophrenia, then MI-therapists would be able to optimize their execution of MI.

**Table 1:** Factors involved in psychological interventions^a^.

**Clinician factors**: what the clinician does in the treatment: behaviors, directives, characteristics. **Client factors**: what the client does in treatment: behaviors, verbalizations, characteristics. **Mechanisms of change**: the processes that emerge from the clinician and client factors that explain how these active ingredients lead to change. **Active ingredients**: the specific ingredients in the intervention that cause the change.

a Based on Nock (15).

In a previous study, we focused on the patient process in MI and we found three factors for successful MI in patients with schizophrenia: a trusting relationship between patient and therapist, the therapist's ability to adapt the MI-strategy to the patient's process, and relating the patient's values to long-term medication adherence (24). In the current study, we focus on therapist strategies to effectively employ MI, i.e., if and how the therapist applies active ingredients, and whether these stimulate mechanisms of change.

## Methods

### Aim

The aim of this study is to explore which clinician factors are employed by MI-therapists, and whether these clinician factors activate client factors, and whether this triggers hypothetical mechanisms of change.

### Study Population

The cases were the audiotaped and transcribed MI-sessions of 14 patients who participated in the intervention group of a Dutch randomized controlled trial (RCT) on MI to promote medication adherence in patients with schizophrenia (4). All patients had recently experienced a psychotic relapse after nonadherence to treatment with antipsychotic medication. Some patients received inpatient psychiatric treatment during all MI-sessions. Other patients received inpatient psychiatric treatment at the start of the MI-intervention, and were dismissed from this treatment in the course of the intervention, and further received treatment from outpatient facilities. For other patients, the complete intervention was executed while receiving community mental health care. The mean age of the patients was 35.5 year (range: 23–48). Four patients were female. Two patients had primary education or less, ten patients had secondary education, and two patients had tertiary education or further education. The mean duration of their mental illness was 6.9 years (range: 1–23). The DSM IV diagnoses were schizophrenia (ten patients) or schizoaffective disorder (four patients).

With the patient's consent, the MI-sessions in the original RCT were audio-recorded. The five therapists (a psychiatrist, three community mental health nurses, and a psychologist) were not involved in the regular treatment of the patients. Before the study, the therapists had no previous experience in MI and they followed a 32-h training by a certified MI-trainer. All MI-therapists participated in monthly supervision on MI-fidelity.

### Mixed Methods

We used mixed methods to study if and how MI-therapists apply clinician factors to activate client factors, and, through these, stimulate hypothetical mechanisms of change (after this: mechanisms of change).

First, to find potential active ingredients and mechanisms of change in MI, we performed a literature search in PsycInfo and in PubMed (search string 1: “motivational interviewing” and “active ingredients”; search string 2: “motivational interviewing” and “mechanism* of change”) and in textbooks on MI (e.g., 14, 16). We also searched for relevant cross-references in the reference list in the selected articles.

Next, we performed a qualitative multiple case study (25) to explore clinician factors, client factors and mechanisms of change in the process of MI. This design contains three phases: single case analysis, cross-case analysis, cross-case synthesis (25). The single case analysis was an analysis of every case separately, guided by worksheets with questions on which the analysis focused. In the cross-case analysis, the findings from the separate cases were merged into clusters. In the cross-case synthesis, these clusters were translated in cross-case assertions, and the evidence for these assertions was reviewed.

In addition, we used sequential analysis (26) to find the probabilities that specific therapist use of MI-techniques, such as a reflection, is subsequently followed by patient change talk or patient sustain talk.

### Data Collection and Analysis

To be included, cases had to have at least three audiotaped sessions. We excluded patients with severe psychotic symptoms which hindered effective communication and participation in the MI-sessions. Patients with moderate psychotic symptoms, who were able to effectively participate in the MI-sessions, were not excluded.

The audio recordings were transcribed and parsed in patient and therapist utterances in accordance with the coding manuals of the Motivational Interviewing Skill Code 2.1 (MISC 2.1) (27) and the Motivational Interviewing Sequential Code for Observing Process Exchanges (SCOPE) (28). We used MISC 2.1 Global Ratings (7-point scores) to score the therapist behavior on three dimensions (acceptance, empathy, MI-spirit), and to score the level of patient self-exploration. The SCOPE was used to sequentially code the patient and the therapist communication behavior in 20 codes for the therapist, and 10 codes for the patient language (**Table 2**) (29). Also, we computed five summary scores as suggested in the coding instruments, to assess the therapist fidelity to MI and thus the quality of the MI delivered. After a 37-h training, two coders coded all MI-sessions [for details, see Dobber et al. (24)]. A random selection of 10% (n = 7) of the sessions were re-coded by the same coder to verify intra-rater agreement, and another randomly selected 20% of the sessions (n=13) were double coded by the two coders independently, to compute the inter-rater agreement. For the global ratings, we considered a maximum of one-point difference on the 7-point scales as an agreement, and a difference of more than one point as a disagreement. So, we dichotomized the scores to “agreement” and “disagreement”. For the intra-rater agreement, we found a Kappa of.77 for the behavior codes, and a Kappa of 1.0 for the global ratings. For the inter-rater agreement, the Kappa's were.71 and.84, respectively.

**Table 2:** Codes for therapist and patient verbal behavior.

	Codes
Therapist behavior	advise with permission, advise without permission, affirm, confront, direct, emphasize control, facilitate, feedback, filler, general information, opinion, permission seeking, question, raise concern, reflect, self-disclosure, structure, support, warn, not encodable
Patient behavior	ask, follow/neutral, commitment, desire, ability, need, reasons, taking steps, other, not encodable

Based on SCOPE (28).

While performing the multiple case study analysis, the first author (JD) produced a detailed log on the findings and the decisions during the research process. Furthermore, in accordance with the method of multiple case study analysis (25), the analyst used worksheets to perform a systematic analysis and to register the findings, and composed detailed case reports. The worksheets concentrated on:

- how clinician factors interacted with the client factors,
- the hypothetical active ingredients, used by the MI-therapists,
- clues for the stimulation of which mechanisms of change, and
- how the MI-therapist applied the active ingredients within the four MI-processes (engaging, focusing, evoking, planning).

For the latter, we constructed a worksheet based on the targets of MI-consistency in the Motivational Interviewing Target Scheme 2.1 (MITS 2.1) (30, 31). In addition, based on the textbook by Berger and Villaume on MI for health care professionals (32), we added the concept “sense making” (see **Table 3**). This concept refers to the phenomenon that patients develop their own ideas and beliefs about what is happening to them (for instance their illness) and how they should cope with what they perceive is happening to them. These beliefs explain the patient's stance towards therapy and consequently to using or not using medication. The therapist needs to understand this patient perspective to effectively apply the clinician factors and strengthen the patient's motivation for medication adherence [see also Berger and Villaume (32)]. Two investigators (BvM and CL) checked all these steps, and, for quality assurance of the research process, independently chose a subset of these materials and performed an inquiry audit. To check the reliability of the findings, another independent investigator double analyzed two cases. In case of disagreement we checked the original data to resolve the disagreement.

**Table 3:** Topics of a worksheet for the qualitative analysis of the cases.

Target	Description
1. Activity emphasis	The therapist chooses to perform the activity that, at any particular point of the conversation, contributes most to behavioral change.
2. Posture, empathy and collaboration	The therapist engages with the patient and demonstrates accurate understanding of the patient's perspectives and feelings, and works with the patient in a purposeful collaboration.
3. Independence	The therapist emphasizes the patient's control over his/her decisions and behavior, and encourages the patient to take responsibility for his/her decisions and behavior.
4. Evocation	The therapist elicits patient change talk and elaborates on this. Also, the therapist softens the patient's sustain talk.
5. Navigation	The therapist ensures that the conversation progresses in the direction of the change goal.
6. Contrasts	The therapist supports the patient to relate the target behavior to his/her values and life goals, and may develop discrepancy between values, goals and present behavior.
7. Structured brief tactics	The therapist performs optional MI-components as conversational strategies as short routes to facilitate the patient's process. Examples of these tactics are the use of “importance rulers”, “confidence rulers”, “a typical day”, and the composition of a “change plan”.
8. Information and advice	The therapist gives only information and advice after (implicit or explicit) permission of the patient, and in an effective way.
9. Sense making	The therapist actively tries to understand the patient's perspective on his/her health problems and the target behavior, and tries to influence the patient's sense making.

Topic 1 to 8: based on the MITS 2.1 (30).

Topic 9: based on Berger and Villaume 2013 (32, 33).

Finally, we used the Generalized Sequential Querier (GSEQ 5.1, software for analyzing sequential observational data) (26, 33) to perform a sequential analysis. Thus, through GSEQ 5.1, we computed the probability that a certain patient motivational statement (e.g., change talk), immediately followed any specified therapist verbal behavior (e.g., an open question querying the target behavior) within the MI-sessions. The p-values for the probabilities resulting from the sequential analysis, were not corrected for multiple analyses. Because of the low frequency of some verbal behavior codes, we combined these codes in broader categories on the basis of MI-theory (14, 16) and previous research (34, 35). For patient verbal behavior, we composed three categories: Change talk, Sustain talk, and Neutral. Apart from Reflections and Questions, we created three categories for the therapist verbal behavior: Sequential MI-consistent behavior, MI-inconsistent behavior, and Other.

## Results

### Development of the Model of Active Ingredients and Mechanisms of Change

**Figure 1:**
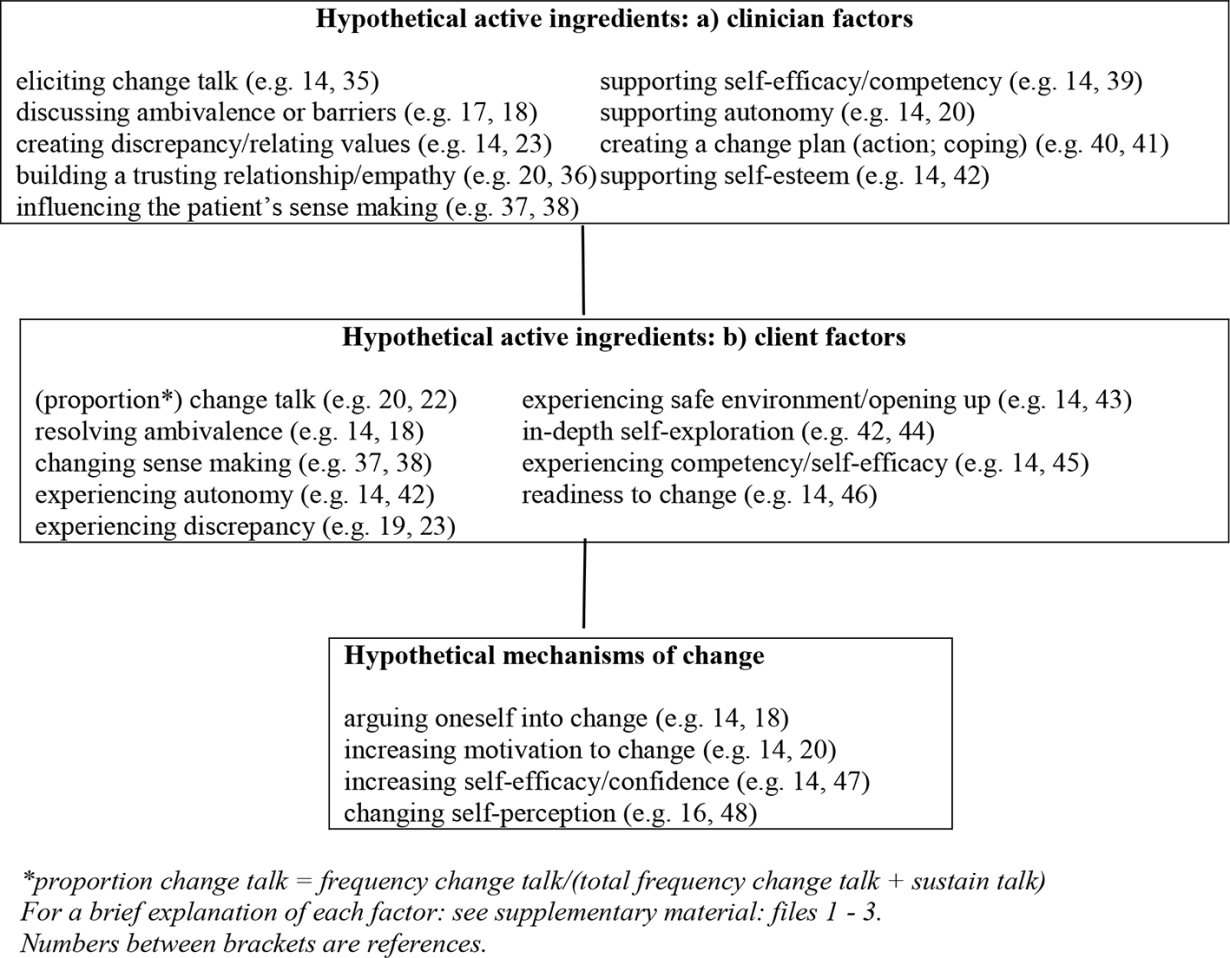
Model of hypothetical active ingredients and mechanisms of change in MI for medications adherence in patients with schizophrenia. *Proportion change talk = frequency change talk/(total frequency change talk + sustain talk). For a brief explanation of each factor: see **Supplementary Material: File 1****–****3**. Numbers between brackets are references.

The composition of the model is based on both the literature search and MI-textbooks [e.g., (14, 16)]. Our literature search yielded 89 articles, of which, based on title and abstract, the full text of 33 articles were retrieved. Of these, nine articles were excluded because of lack of relevance for determining potential active ingredients or mechanisms of change. As a result, we used 24 articles and four textbooks to compose our model of hypothesized active ingredients and hypothesized mechanisms of change (**Figure 1**).

### Included Cases

There were 16 cases with three or more audiotaped MI-sessions. We excluded two cases with patients presenting with active psychotic symptoms during the MI-sessions, since this made practicing MI impossible. So, 14 cases, comprising 66 audiotaped MI-sessions were included. One therapist performed MI in five cases (28 sessions), one therapist performed MI in four cases (19 sessions), two therapists each performed MI in two cases (eight sessions per therapist), and one therapist performed MI in one case (three sessions).

### Which Clinician Factors Are Present?

Overall, eight out of nine clinician factors (see **Figure 1**) were applied by the therapists. There was great diversity among the therapists in the number of clinician factors applied (**Table 4**). The most frequently used clinician factor was “eliciting change talk”, without which the intervention would not be MI (12). Still, in two cases the interaction with the patients and the course of the sessions hindered the therapist to elicit change talk. There was some change talk, but in these sessions it was of such poor quality or artificially elicited (e.g. “You’re sleeping well, aren’t you, on these medications?”), that we did not consider it as a potential active ingredient. In the first case, the patient avoided serious conversations about medication adherence, and in the second case, a trusting relationship could not be established. This was apparent from superficial conversations with limited openness shown by the patient. A trusting relationship is fundamental to MI, and in this case the conversations, which were also strongly influenced by a language barrier, were dominated by mutual misunderstandings. Hence, though all therapists showed “empathy”, not all therapists succeeded to always establish a ‘trusting relationship’ (**Table 4**).

**Table 4:** Application of clinician factors.

Hypothetical clinician factors	Frequency	Number of therapists (n=5) who applied it	Number of clients (n=14) it was applied to
Eliciting change talk	61	5	12
Building a trusting relationship/empathy	*	4	10
Supporting self-esteem	10	4	6
Discussing ambivalence and/or barriers	7	3	3
Influencing the patient's sense making	6	3	4
Supporting self-efficacy/competency	16	3	4
Supporting autonomy	7	3	5
Creating discrepancy/relating values	9	3	4
Creating a change plan	0	0	0

*Mostly applied and maintained through all sessions.

### Which Client Factors Are Activated by the Clinician Factors?

Except for “readiness to change”, we observed all client factors from our model (**Table 5**). Often, a clinician factor activated a variety of client factors, sometimes simultaneously. Discussing ambivalence, for instance, may activate “patient change talk”, but can also activate the “patient experiencing discrepancy” and can lead to “resolving ambivalence”. The application of a clinician factor however, does not always activate the targeted client factors. While “eliciting change talk” (almost) always led to “change talk”, “supporting self-efficacy” activated only in 25% of the applications a client factor (**Table 5**, see **Box 1** for a successful and a less successful example).

**Table 5:** Clinician factors, client factors, mechanisms of change.

Clinician factors	Frequency	Client factors	freq	Hypothetical mechanisms of change	Frequency
Building a trusting relationship/Empathy	*	Experiencing safe environment/opening up	*		
		In-depth self-exploration	*		
Eliciting change talk	61	Change talk Sustain talk** Experiencing competency/self-efficacy Experiencing autonomy	60 10 1 1	Arguing oneself into change	12
Supporting self-esteem	10	Experiencing competency/self-efficacy Experiencing autonomy	1 1	Changing self-perception Increasing motivation to change	1 1
Discussing ambivalence and/or barriers	7	Experiencing discrepancy Change talk Sustain talk** Resolving ambivalence	1 5 2 2	Arguing oneself into change Arguing oneself into change	1 1
Influencing the patient's sense making	6	Changing sense making Resolving ambivalence	2 1	Arguing oneself into change Arguing oneself into change	1 1
Supporting self-efficacy/competency	16	Experiencing competency Experiencing autonomy	3 1	Increasing motivation to change	1
Supporting autonomy	7	Experiencing autonomy	3	Increasing motivation *not* to change***	1
Creating discrepancy/relating values	9	Change talk Changing sense making Experiencing discrepancy Resolving ambivalence Experiencing autonomy	1 2 1 1 1	Arguing oneself into change Arguing oneself into change Increasing motivation to change	2 1 1

*Mostly applied and maintained through all sessions.

**Sustain talk is a client factor in favor of nonadherence.

***This patient did not feel ambivalent about his decision to stop the medication as soon as possible.

**Box 1:** Examples of Supporting self-efficacy and patient reaction.

Therapist: “And you are good at that: fine-tuning your medication dose, you are able to do that yourself.” Patient: “Yes, I guess 10 years of experience made me some kind of an expert by experience.” (Case 5) Comment: the patient experiences the therapist emphasizing his control over his medication as an affirmation of his competence.
Therapist: “So you do see which factors throw you off-balance and which, in contrast, keep you stable: your medication use, on which you have a clear vision of now, and alcohol-use which you want to, and can, control. And also, regularity in your life and daytime activities.” Patient: “Yes.” (Case 14) Comment: the summarizing character of this supporting reflection seems to restrict the effect of the clinician factor ‘Supporting self-efficacy'.

### Do Client Factors Lead to Mechanisms of Change?

Since mechanisms of change refer to processes within the patient's mind, it is not possible to observe these psychological processes from an outsider perspective. One can listen to the patient's change talk, and infer from the content and course of the patient change talk that he/she is arguing him or herself into change, but one cannot be certain this process is actually happening [see also Miller and Rollnick (18)]. So, when listening to motivational interviewing sessions, we needed to confine ourselves to, based on the content of patient speech, recognizing clues of a psychological process which might take place within the patient.

We recognized clues for mechanisms of change in sessions with six out of 14 patients. Clues for the mechanism of change “arguing oneself into change” were most prevalent, and the client factor that mostly preceded it was “change talk” (**Table 5**, see **Box 2** for an example). However, this may paint a slightly distorted picture. While client factors are often activated by the immediately preceding clinician factors, the mechanisms of change are mostly the result of a much longer part of the session and preceded by a sequence of clinician factors and client factors.

Box 2 **Example of stimulation of a mechanism of change: arguing oneself into change.**

Therapist: The medication taking in itself…

Patient: Is no problem.

T: You just think “that's how it is…”

P: Yes.

T: … or “I need it…”

P: Yes, you just accept it … To others, sometimes I tell them to stay on their medication. You know, sort of… (laughs) as if I have to advise them. It's just … to young people I sometimes say: you have to stay on medication, because they think ‘I'm doing fine', you know, what they don't know … But one may have a chronic condition, and the other doesn't. But I have a chronic condition, so I know for the rest of my life I'll have to…

T: How do you see your condition? Sometimes, you experience psychosis, how would you call it? Some people would say schizophrenia, others…

C: With me, they say it's schizoaffective. (…)

T: Do you think you have an illness?

P: Yes. Yes, now, when I use my medication, I'm not ill, obviously. But if I don't use them then I'm ill. I can see that difference, yes.

(Case 1)

The mechanism of change ‘increasing motivation for change’ seemed to occur in non-ambivalent patients who were arguing to strengthen their decision pro or against long-term medication adherence. The clue for “changing self-perception” was observed in a session in which the patient at first presented himself as “someone who *knows* that medication works”. After therapist's reflection on *understanding* the importance of medication and the affirmation on the patient's *insight*, the patient expressed being “someone who *understands the utility* of medication”, thus fostering a self-perception which may strengthen his medication adherence. We did not find clues for the stimulation of the mechanism of change “increasing self-efficacy/confidence”.

### How Does the MI-Therapist Apply Active Ingredients to Influence Mechanisms of Change?

#### Quantitative Analysis

The sequential analysis [in GSEQ 5.1 (26, 33)] over all 66 MI-sessions shows that the client factor “*Change talk*” is usually elicited by reflections directed at medication adherent behavior or intentions (Reflection+) and by questions directed at medication adherent behavior or intentions (Question+). *Sustain talk* is mainly elicited by reflections directed at medication non-adherent behavior or intentions (Reflection-). Surprisingly, sequential MI-consistent (sMI-consistent) therapist behavior like Affirmation and Emphasizing Control, was nine out of ten times followed by a neutral client statement, while we expected a higher proportion of change talk (**Table 6**). We performed a sensitivity analysis omitting the sessions of one patient in which a language barrier possibly hampered the MI-conversations. The sensitivity analysis revealed minor differences in some of the probabilities. In our opinion, this does not affect the interpretation of the analysis. In the **Supplementary Material, File 4**, we present the results of the sensitivity analysis.

**Table 6:** Conditional probabilities^ab^.

Target (patient statements; n=6269) Given (therapist statements; n=6474)	Sustain talk^c^	Change talk^d^	Neutral^e^
Other^f^	.06	.07	.87
2-sided-question (±)^g^	.19	.36	.45
Question-	.58	.08^*^	.35
Question neutral	.01	.02	.96
Question+	.04	.69	.27
2-sided reflection (±)^g^	.24	.29	.47
Reflection-	.67	.05	.29
Reflection neutral	.01	.01	.98
Reflection+	.02	.74	.24
sMI-consistent^h^	.04^*^	.06	.90
MI-inconsistent^i^	.04^*^	.07^*^	.90

a Probability of a certain type of patient statement given a particular type of therapist statement.

b All: p ≤ 0.01, except ^*^0.01< p < 0.05.

c Sustain talk comprises desire to change, ability to change, reasons to change, need to change, commitment to change, taking steps to change, and other pro-change statements.

d Change talk comprises desire not to change, ability not to change, reasons not to change, need for status quo, commitment to status quo, taking steps to status quo, and other counter-change statements.

e Neutral comprises ask, follow/neutral, and not encodable patient statements.

f Other comprises facilitate, filler, self-disclosure, general information, raise concern, structure, advising with permission, not encodable.

g 2-sided means questions or reflections addressing both change talk and sustain talk

h sMI-consistent, sequential MI-consistent, and comprises affirmation, emphasizing control, permission seeking, offering support.

i MI-inconsistent comprises confrontation, directing, warning, giving opinion, advising without permission.

Note that row percentages add up to 100 (except for rounding).

#### Qualitative Analysis

Below, we describe the application of the active ingredients in the four MI-processes: engaging, focusing, evoking, and planning.

##### Engaging

Though posture, empathy and collaboration remained important through all sessions, the clinician factor “trusting relationship”, was built in the first session. Making an effort to understand the patient's perspective, showing empathy and interest in the patient and his/her story established rapport, which was maintained through all sessions. Moreover, therapists who understood how the patient made sense of his/her psychoses and of his/her antipsychotic medication treatment were able to use the clinician factor “influencing the patient's sense making” at a later moment in the evoking process of the MI-sessions (**Box 3**).

Box 3 **Influencing the patient’s sense making.**

This patient wants to have control over her life, but her life is negatively impacted every time she experiences a psychosis. She thinks that medication is helpful to recover from psychosis. However, during stable periods, she finds only a low dose of medication acceptable, or no medication at all. She prefers no medication because of the drugs' side effects and she feels more autonomous without medication.

Therapist: So, what I learn from you is that in your opinion medication may be a decisive factor to remain stable.

Patient: Yes, if it is not, that would be a problem, what else could I do then?

T: And you mentioned that if things go wrong, and you were off medication for a longer period of time, things seem to get worse.

P: Yes, it does.

T: Is that also a consideration?

P: It is, yes, it is. It may go well for say 3 months, but I've learned from the past that it ends up going wrong. So, medication should be used wisely, I should not experiment with it. Although I'm still a little bit troubled with the physical side effects for which I also need to see an internist, how many sorts of medication do I have to take to stay stable?

T: These long-term consequences are a concern for you…

P: They are.

T: …and at the same time it is obvious for you that medication protects you.

P: It is. (…) Apparently, I do need medication after all … I think.

(Case 14)

##### Focusing

In most sessions, the therapist managed to focus on the target behavior of medication adherence. However, therapists who were able to consistently select the conversational activity (e.g., active listening, goal setting, exploring ambivalence, providing information) which fit best to the patient's motivational process, used a higher variety of clinician factors to activate client factors.

##### Evoking

The quality of evocation of change talk varied between therapists, and for some therapists this variation also appeared within the sessions. Good quality “change talk” (in terms of depth, amount and strength) mostly occurred as a result of an MI-strategy in which the therapist navigated to support the patient to ‘resolve his ambivalence’, or to “develop discrepancy” (**Box 4**). However, the fine line between evocation of good quality change talk and lower quality change talk is easily crossed. Sometimes poor quality change talk was elicited, in particular when the therapist artificially sought to elicit change talk without embedding this in a more comprehensive MI-strategy (T: “Why is medication important for you according to your physician, do you know?” P: “No, just for my illness.” T: “Yes, for your illness. So it does help you.”).

Box 4 **Evoking change talk.**

Patient: I stopped taking my medication because I thought … I feel fine … I'll quit taking them…

Therapist: I'm cured.

P: But that's what the medication does.

T: What does the medication do?

P: Make you feel better. So, if you feel fine, you should not stop taking medication but just continue … that's what the medication does.

T: You have experienced that, you learned from that.

P: I did. If I stop taking my medication that will make the chance of relapse much larger than when I do take my medication.

T: Did other persons tell you this, or do you feel … experience that this is how it works?

P: Yes, I've noticed that it works like this.

(Case 12)

**Figure 2:**
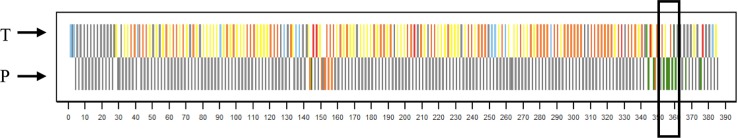
Visual Visual overview of session 1, case 12. Overview of sequentially coded session. Colored bars therapist verbal behavior: dark green = question querying for change, or two-sided question; light green = reflection of change talk, or two-sided reflection; yellow = question querying counter-change, or question not directed at target behavior; orange = reflection of sustain talk, or reflection of neutral talk; blue = sequential MI-consistent techniques (affirm, emphasize control, permission seeking, support); red = MI-inconsistent techniques (confront, direct, warn, opinion, advice without permission); gray = other techniques. Colored bars patient verbal behavior: green = change talk; orange = sustain talk; gray = neutral. On the axis, the sequential utterance number is displayed. The line in the black rectangle shows the session part displayed in **Box 4**.

Since change talk plays a central role in MI (12, 14, 16) and as it is considered as an essential part of MI (12), it may be one of the most important client factors. To gain more insight in the pattern of change talk during the sessions, we added a visual overview of a MI-session (**Figure 2**), focused on occurrence of change talk and sustain talk, and the applied therapist techniques (for a visual overview of all 66 MI-sessions, see **Supplementary Material: File 5**).

“Developing discrepancy” is an important MI-strategy, especially with medication-adherence as target behavior, since many patients with medication-nonadherence in the recent history do not consider medication-use in the remission state as desirable or in line with their values and life goals. Values and life goals may provide, however, powerful motives to change the patient’s perspective on long-term medication-adherence (24). Autonomy and independence are important values related to medication adherence, as pointed out by four patients, and these patients felt that the need for medication restricts their autonomy and independence. Only a few therapists addressed this topic to discuss if and how medication may contribute to autonomy and independence. Especially if patients expressed their intention to stop using medication in the near future, therapists tended to argue for medication-adherence instead of accepting the patient’s perspective at that moment, thus taking over the responsibility and reducing the patient’s independence (**Box 5**).

Box 5 **Arguing for medication adherence.**

Patient: If I can take care of my own things, then I won't collect my medication at the clinic anymore, because previously I didn't go to the clinic for medication.

Therapist: Later, when you have a job, do you think that you'll need the medication and collect it somewhere else, or will you stop taking medication?

P: Yes, I'll stop taking medications. It is not a good thing to take medications for your whole life, but just for 3 years like I have done now. Previously I didn't take medication, and it's no good to be tired and fat. (…) If I have a job, no one can force me to take medications.

T: So, if you are not dependent anymore, there is no obligation for you to come to the clinic.

P: Yes.

T: Earlier you told me you don't think taking medication is a problem. And your mother thinks that it is very important for you to use medication.

P: Yes.

T: Will it cause big problems for you later?

P: No, when I have a home of my own, no one can say anything about that.

(Case 3)

Some therapists used a decision balance (listing the pros and the cons of medication adherence), which was helpful when the therapist listened well to the patient and reflected his/her concerns, and when the therapist elaborated on the pro-side of medication use. However, often, the performance of a decision balance happened at the cost of much sustain talk. Giving information and advice is another technique that differentially could either support patient engagement and the patient’s motivational process, or cause disengagement. Information and advice deepened the conversation if it was tailored to the patient process, or asked for by the patient. But otherwise, it could emphasize the therapist’s expert role and threaten the patient’s feeling of competence and autonomy, and a few times this caused some discord and patient disengagement.

##### Planning

In some sessions therapists and patients discussed potential barriers for prolonged medication adherence and relapse prevention. None of the patients, however, created a “change plan” or a relapse prevention plan.

##### Therapist Fidelity

The quality of the MI delivered by the therapists also influenced the appearance and the potency of the active ingredients. As shown through the five summary scores of the MISC (27) and the SCOPE (28), the therapists performed MI at beginning proficiency level (**Table 7**). Overall, the therapists were good at verbalizing complex reflections, but were inclined to ask closed questions. One therapist focused mainly on factual information, and tended to pursue his own agenda, with limited effort to gain a deeper understanding of the patient’s perspective and experiences.

**Table 7:** Therapist fidelity ratings.

Therapist	Global Therapist Ratings^a^	Reflection/Question ratio^b^	Proportion open questions of all questions asked^c^	Proportion complex reflections of all reflections^d^	Proportion MI- consistent behavior^e^
**1**	+	+	–	++	+
**2**	+	–	–	++	+
**3**	–	+	–	++	+
**4**	+	–	+	++	+
**5**	+	–	–	++	+

Scores are means over all of the therapist's sessions.

-=not proficient; +=beginning proficiency; ++=competent [based on thresholds in manuals (27, 49)].

a Global Therapist Ratings. Scores based on mean ratings on three 7-point Global Rating scales (Acceptance, Empathy, MI-Spirit) (27). Threshold beginning proficiency: mean rating = 4.9 (49).

b Reflection/Question ratio. Ratio between reflections and questions (27). Threshold beginning proficiency if R:Q=1 (49).

c Proportion open questions of all (open and closed) questions (27). Threshold beginning proficiency if %OQ=50% (49).

d Proportion complex reflections of all (simple and complex) reflections (27). Threshold competency is %CR=50% (49).

e Proportion MI-consistent behavior of MI-consistent and MI-inconsistent behavior (27). Threshold beginning proficiency if %MICO=90% (49).

## Discussion

This study was designed to study the mechanisms of MI for medication adherence in patients with schizophrenia. We unraveled the MI-intervention in active ingredients (clinician factors and client factors) and mechanisms of change, and we systematically studied the application of active ingredients and the appearance of clues for mechanisms of change in 66 MI-sessions with the target group. Our model helped us to see “MI at work”. It offered a view on how therapists act to influence the patient’s behavior, activating client factors, which may sometimes stimulate the occurrence of mechanisms of change: covert assumed psychological processes that are associated with a subsequent change in medication adherence [see also Miller and Rollnick (18)].

We found that whether the clinician factor activated one or more client factors depended on the specific clinician factor as well as whether or not the clinician factor was embedded in a broader MI-strategy. In a few sessions, the therapist was not able to apply such a strategy, and in these sessions therapists sometimes elicited change talk in an artificial way. This resulted in poor quality change talk, which never led to an active ingredient. These practices, however, occur in particular in newly starting MI-therapists at beginning proficiency.

We also detected indications for the appearance of three of the four mechanisms of change of our model: “arguing oneself into change”, “increasing motivation to change”, and “changing self-perception”.

We did not observe the clinician factor “creating a change plan”, the client factor “readiness to change”, and the mechanism of change “increasing self-efficacy/confidence”. The construction of a change plan was not included in the manual of the intervention (50) in the RCT (4) from which the MI-sessions originate. This may explain the absence of the factors “creating a change plan” and “readiness to change”. It may also be an explanation for the absence of the mechanism of change “increasing self-efficacy/confidence”. In four cases the therapists supported existing self-efficacy, but in none of the cases the therapist addressed the increase of self-efficacy in the context of creating a change plan for medication adherence.

Most of the present knowledge about active ingredients and mechanisms of change in MI originates from alcohol dependency research [e.g., (17, 19, 21, 22)]. Magill et al. (21) used mediation analysis to test a model with active ingredients, mechanisms of change and patient outcomes in a brief motivational interviewing intervention in heavy drinking underage young adults. Despite the differences between the studies in target populations, target behavior, and study design, two out of three of the MI-specific mechanisms of change of the model by Magill et al. (21), were also found in our study (“experiencing discrepancy”, which we consider an active ingredient, and “increasing motivation for change”), but we did not find “increasing self-efficacy” in our sample.

In contrast to Magill et al. (21) our model differentiates between clinician factors and client factors, consistent the description by Nock (15). The influence of client factors in psychological interventions is recognizable in MI, since the mere act of “eliciting change talk” does not stimulate a mechanism of change. It depends on the client reaction (e.g. change talk in a certain depth, amount and strength) whether a mechanism of change is stimulated. Moreover, in our qualitative analysis we found that mechanisms of change mostly are a result of a MI-strategy adapted to the patient process, which comprised an interaction between therapist and patient during larger session parts, and included a variety of clinician factors and client factors. Also, while interaction between clinician factors and client factors seems to be a prerequisite for the appearance of a mechanism of change, many of these interactions did not result in a stimulation of a mechanism of change.

Kazdin and Nock (51) point out that knowing how or why psychological interventions work presumes knowledge about necessary and sufficient ingredients, effective and non-effective doses, and factors impeding change. Our study suggests that in particular the client factors are in fact a pool of factors from which, if properly activated by clinician factors, different combinations can form active ingredients that stimulate a mechanism of change.

However, a mechanism of change for a specific outcome is only a mechanism of change if it causes that specific outcome. We did not study the relation between the mechanisms of change and medication adherence. Before studying such a relationship, we first needed to know what active ingredients are actually delivered in the intervention under study, and whether there are sufficient clues for the stimulation of mechanisms of change by the active ingredients. For causality, statistical mediation is required (15) in addition to the causal guidelines [e.g., (15, 51, 52)]: strong association, specificity, gradient/dose-response relationship, temporal relation, consistency, experiment, plausibility and coherence. Of these, we only showed temporality, and we had to accept the plausibility of the mechanisms of change from the theory of MI (14, 16).

### Limitations and Strengths

A limitation of this study is the limited visibility or measurability of most of the client factors and mechanisms of change, and the subsequent interpretative character of the findings. Furthermore, we studied only a small sample of 14 patients. However, we believe that the sample was pragmatic and population-based, and in our opinion representative for schizophrenia patients with recurrent psychotic episodes, medication non-adherence, and frequent re-admission. In addition, due to the rigorous (systematic and transparent) method and the strict quality control measures, we believe the findings are credible and trustworthy.

Our tentative model is based on MI-theory and research literature, thereby reflecting the current state of the MI-knowledge on this subject. In spite of this, the hypothetical character of our model of active ingredients and mechanisms of change is still a limitation, and there may be other, possibly unknown, factors or mechanisms that are missing in the model (53, 54).

A strength of this study is the depth of analysis. We analyzed beyond the MI-measurement instruments [MISC (27), SCOPE (28), MITS (30)], and used both quantitative and qualitative research methods. This thorough analysis enabled us to study the interactions between ingredients and mechanisms. A better understanding of this is an important step in the development of knowledge on MI. With the results of this study, we add a building block to answer the question how and why MI works in general, and particularly how MI works in patients with schizophrenia with medication adherence as target behavior.

## Conclusions

A large variation in the application of clinician factors enables the therapist to build a MI-strategy. The clinician factors activate the client factors, of which in our data ‘change talk’ was the most prevalent. It is plausible, however, that it is not about individual clinician factors activating individual client factors, but about a sufficient combination of factors. This combination acts as an active ingredient and can trigger a mechanism of change.

The most important conversational techniques that shape the clinician factors we observed are reflections and questions addressing medication adherent behavior or intentions, often followed by the client factor “change talk”. “A trusting relationship and empathy” turned out to be an important clinician factor, that enabled both therapist and patient to attain sufficient depth in the conversation through which clinician factors and client factors allow for a fruitful interaction with opportunities to trigger mechanisms of change.

Our model enabled us to see “MI at work”, and formed a basis for qualitatively studying MI. The model and our findings may help practitioners to improve the effectiveness of their MI-strategies to a more effective MI, in which active ingredients are intentionally employed to increase the probability of behavior change.

MI may be more effective if the therapist is informed about the active ingredients and the mechanisms of change. The current study provides possible ingredients of effective patient-therapist interactions triggering mechanisms of change. However, whether these mechanisms lead to better outcomes needs to be studied in further detail. A next step in research may be to study whether there are better outcomes for patients with MI-sessions in which one or more mechanisms of change appeared, compared to patients for whom no mechanisms of change were observed.

## Data Availability Statement

Parts of the dataset generated and analyzed for this study can be found in the Figshare repository: https://doi.org/10.21943/auas.9505436. Parts of the datasets generated and analyzed during the current study are not publicly available due to identifying patient information. Data may be available from the corresponding author upon request, but restrictions apply on the availability of these data in accordance with the ethical rules of the Medical Ethical Committee of the Amsterdam UMC.

## Ethics Statement

The studies involving human participants were reviewed and approved by the Medical Ethics Committee of the Amsterdam UMC, University of Amsterdam. The patients/participants provided their written informed consent to participate in this study. Written informed consent was not obtained from the individual(s) for the publication of any potentially identifiable images or data included in this article.

## Author Contributions

JD, CL, BM, GR, EB, RP, WS, and LH contributed to the study design. JD and EB performed the data acquisition, JD, GR, and EB performed the data analysis. JD, GR, EB, and LH interpreted the data, and CL and BM checked the data-interpretation. JD, CL, BM, GR, EB, RP, WS, and LH participated in writing the manuscript. All authors approved the final manuscript.

## Funding

This work was supported by a research grant from the Netherlands Organization for Scientific Research (NWO, grant number 023.004.060) to JD.

## Conflict of Interest

The authors declare that the research was conducted in the absence of any commercial or financial relationships that could be construed as a potential conflict of interest.
