# Supplementary material for: Active Ingredients and Mechanisms of Change in Motivational Interviewing for Medication Adherence. A Mixed Methods Study of Patient-Therapist Interaction in Patients With Schizophrenia
Source: Front Psychiatry. 2020 Mar 24;11:78. doi: 10.3389/fpsyt.2020.00078 (PMC7105777; doi:10.3389/fpsyt.2020.00078)
Supplement: Supplementary file 1 [file DataSheet_1.pdf]

Supplementary material file 1. Clinician factors ("what the clinician does in the treatment, including clinician behaviors, characteristics, and directives" Nock, 2007, p.8s [1]).

Eliciting change talk
The therapist purposefully employs activities to elicit change talk (e.g. through complex reflections or open ended questions). Change talk entails patient statements in favor of behavior change in the direction of the target behavior: long-term medication adherence.

Discussing ambivalence and/or barriers
The therapist openly/explicitly or implicitly talks about the patient's ambivalence towards medication adherence. The therapist may discuss both sides of the ambivalence, or how the ambivalence may be solved, or differentially simply reflect the patient's concerns on the contra side and reflect and elaborate on the pro-side of change.
The discussion of barriers may concentrate on identifying barriers and on discussing the patient's concerns about these barriers.

Creating discrepancy / relating values
The therapist tries to direct the course of the conversation in such a way that the patient relates his/her own values or life goals to the target behavior (long-term medication adherence). In doing so, the therapist may attempt to create discrepancy, i.e. the patient experiences a gap between the present situation and the desired situation.

Building a trusting relationship / empathy
Therapist and patient develop a relationship of mutual respect and trust. The therapist takes a listening and empathic stance, shows genuine interest in the patient, and sympathizes with the patient's experiences (and does not pursue his/her own agenda). The patient is being listened to, understood, or the therapist takes an effort to understand him/her.

Influencing patient's sense making
By providing information, the coach attempts to influence the patient's sense making (in which the present behavior [poor medication adherence] seems obvious and logical to the patient) in such a way that the patient finds his/her sense making no longer logical, or even incorrect.

Supporting self-efficacy / competency
The therapist promotes or affirms the patient's experience of competency or confidence or the belief of self-efficacy, e.g. by discussing coping strategies to handle these barriers.

Supporting autonomy
The therapist promotes or affirms that the patient is the only person who decides (about medication adherence); or promotes or affirms that the patient is gaining control or has control over the medication by using it for his/her own purposes and goals; or promotes or affirms the patient's (feeling of) autonomy.

Creating a change plan
The therapist and the patient work out a concrete plan that fits the patient's actions and strategies for long-term medication adherence or to avoid or cope with potential barriers for long term medication use.

Supporting self-esteem
The therapist emphasizes (e.g. affirms or reflects) a positive patient trait or skill.

Reference

1. Nock MK. Conceptual and design essentials for evaluating mechanisms of change. Alcohol Clin Exp Res. 2007;31(S3):4S-12S.
