# Supplementary material for: Active Ingredients and Mechanisms of Change in Motivational Interviewing for Medication Adherence. A Mixed Methods Study of Patient-Therapist Interaction in Patients With Schizophrenia
Source: Front Psychiatry. 2020 Mar 24;11:78. doi: 10.3389/fpsyt.2020.00078 (PMC7105777; doi:10.3389/fpsyt.2020.00078)
Supplement: Supplementary file 2 [file DataSheet_2.pdf]

Supplementary material file 2. Client factors ("what the client does in treatment, including behaviors, characteristics, and verbalizations on their part" (Nock, 2007, p.8s [1]).

Change talk
Patient statements in favor of change: prolonged medication adherence. Patient statements may concern desire, ability, reasons, need, commitment, taking steps, or other pro-change statements.

Resolving ambivalence
The patient expresses a choice for one of the two sides of ambivalence, which resolves the originally present doubt or ambivalence.

Changing sense making
The patient adjusts his/her original explanatory model through which the patient explains his/her medical and psychological condition and his/her coping with it, including his/her sense making of medication (non)adherence.
Or: the patient adjusts his/her reasoning about the consequences of the explanatory model for his/her coping with this condition, including his/her sense making of medication (non)adherence.

Experiencing autonomy
The patient's reaction shows that, due to a therapist statement, the patient experiences autonomy or being in control, in an enhanced degree.

Experiencing discrepancy
Patient's statements show that the patient experiences a gap (or the development of a gap) between the existent situation and the desired situation, recognizing certain life goals or values for which medication adherence can be key to accomplish (a higher degree of) these life goals or values.

Experiencing a safe environment / opening up
The patient overtly talks about his/her concerns and opens up. The patient goes deeper into personal material, spontaneously elaborating on it with feeling.

In-depth self-exploration
The patient explores personally relevant material and may discover new perspectives and/or personal meanings (see also: Client self-exploration, MISC2.1, p.7; 2008 [2]).

Experiencing competency / self-efficacy
The patient reaction shows that, due to a therapist statement, the patient experiences competence or self-efficacy, in an enhanced degree.

Readiness to change
The patient states directly or indirectly to adhere to long-term medication use (while up to that moment, he/she did not completely adhere to long-term medication use).

References

1. Nock MK. Conceptual and design essentials for evaluating mechanisms of change. Alcohol Clin Exp Res. 2007;31(S3):4S-12S.
2. Miller WR, Moyers TB, Ernst D, Amrhein P. Manual for the Motivational Interviewing Skill Code (MISC). Version 2.1. 2008. https://casaa.unm.edu/download/misc.pdf Accessed 27 October 2013.
