# Supplementary material for: Active Ingredients and Mechanisms of Change in Motivational Interviewing for Medication Adherence. A Mixed Methods Study of Patient-Therapist Interaction in Patients With Schizophrenia
Source: Front Psychiatry. 2020 Mar 24;11:78. doi: 10.3389/fpsyt.2020.00078 (PMC7105777; doi:10.3389/fpsyt.2020.00078)
Supplement: Supplementary file 3 [file DataSheet_3.pdf]

Supplementary material file 3. Hypothetical mechanisms of change ("the processes that emerge from or occur as a result of the clinician and client factors, and their interaction, that explain how those factors lead to change in the outcomes of interest" Nock, 2007, p.8s [1]).

Arguing oneself into change
The patient talks about long-term medication use in such a way that he/she convinces him/herself to use medication on a long-term basis (while up to that moment he/she was unconvinced). If the patient was already convinced, he/she may strengthen this belief.

Increasing motivation to change
The patient clearly expresses a stronger motivation for long-term medication adherence than earlier in the same session or in previous sessions.

Increasing self-efficacy / confidence
The patient expresses an enhanced degree of self-efficacy or confidence in his/her ability to adhere to medication on a long-term basis.

Changing self-perception
The patient's statements show a shift in self-perception regarding (an aspect related to) medication use.

Reference

1. Nock MK. Conceptual and design essentials for evaluating mechanisms of change. Alcohol Clin Exp Res. 2007;31(S3):4S-12S.
