# Supplementary material for: Active Ingredients and Mechanisms of Change in Motivational Interviewing for Medication Adherence. A Mixed Methods Study of Patient-Therapist Interaction in Patients With Schizophrenia
Source: Front Psychiatry. 2020 Mar 24;11:78. doi: 10.3389/fpsyt.2020.00078 (PMC7105777; doi:10.3389/fpsyt.2020.00078)
Supplement: Supplementary file 4 [file DataSheet_4.pdf]

Supplementary material file 4. Sensitivity analysis of the conditional probabilities

Conditional probabilities[ab] omitting the sessions of one patient with a language barrier

**Target** (patient statements; n=5954)<br><br>**Given** (therapist statements; n=6122)	**Sustain talk**[c]	**Change talk**[d]	**Neutral**[e]
Other[f]	.05	.07	.88
2-sided-question (±)[g]	.17	.37	.46
Question-	.55	.08**	.37
Question neutral	.01	.02	.97
Question+	.03	.70	.28
2-sided reflection (±)[g]	.23	.30	.47
Reflection-	.64	.05	.31
Reflection neutral	.01	.02	.98
Reflection+	.02	.74	.24
sMI-consistent[h]	.04*	.06	.91
MI-inconsistent[i]	.03*	.07*	.90

[a]*Probability of a certain type of patient statement given a particular type of therapist statement*
[b]*All: $p \leq 0.01$, except *$0.01 < p \leq 0.05$ and **$p=0.08$*
[c]*Sustain talk comprises desire to change, ability to change, reasons to change, need to change, commitment to change, taking steps to change, and other pro-change statements*
[d]*Change talk comprises desire not to change, ability not to change, reasons not to change, need for status quo, commitment to status quo, taking steps to status quo, and other counter-change statements*
[e]*Neutral comprises ask, follow/neutral, and not encodable patient statements*
[f]*Other comprises facilitate, filler, self-disclosure, general information, raise concern, structure, advising with permission, not encodable*
[g]*2-sided means questions or reflections addressing both change talk and sustain talk*
[h]*sMI-consistent = sequential MI-consistent, and comprises affirmation, emphasizing control, permission seeking, offering support*
[i]*MI-inconsistent comprises confrontation, directing, warning, giving opinion, advising without permission*
Note that row percentages add up to 100 (except for rounding)
