# Supplementary material for: Active Ingredients and Mechanisms of Change in Motivational Interviewing for Medication Adherence. A Mixed Methods Study of Patient-Therapist Interaction in Patients With Schizophrenia
Source: Front Psychiatry. 2020 Mar 24;11:78. doi: 10.3389/fpsyt.2020.00078 (PMC7105777; doi:10.3389/fpsyt.2020.00078)
Supplement: Supplementary file 5 [file DataSheet_5.pdf]

Supplementary material file 5. Visual overview of all 66 sessions

The data were collected in the intervention group of a RCT to study the effect of motivational interviewing (MI) to promote medication adherence in patients with schizophrenia (1). All audiotaped MI-sessions were coded using the Motivational Interviewing Sequential Code for Observing Process Exchanges (SCOPE, 2), sequentially coding both therapist and patient communication behavior. Below, 66 MI-sessions are displayed. Both patient verbal behavior and therapist verbal behavior is converted into colors. For reasons of clarity we limited the number of categories for patient verbal behavior to three, and seven for therapist verbal behavior. The categories were composed on theoretical grounds. The therapist behavior is shown through the colored bars in the top half of each figure, while the colored bars in the bottom half show the patient behavior. On the x-axis, the sequential utterance number is displayed. Broadly, the colors green and blue sign “good”, grey signs “neutral”, yellow and orange sign “caution”, and red signs “bad”. For further description of the meaning of the colors: see the legend below. Note that these colors don’t convey the quality and the exact content of the utterances.

In the first sessions of all cases the therapist took some time with the patient and asked him/her to review his/her illness history. Most times, this contributed to the trusting relationship between patient and therapist. This start explains why in session 1, in many cases, the topic of medication adherence played a minor role.

The sequence of the colors shows the course of the change talk and sustain talk, and the therapist techniques preceding and following change talk and sustain talk. The sequence may also reveal interaction patterns. On the basis of MI-theory (3) in good quality MI-sessions, one might expect [1] “green therapist behavior” to be followed by “green patient behavior”; [2] “green patient behavior” to be followed by “green therapist behavior”; [3] none, or just a small number or “red therapist behavior”; [4] a “mix of yellow and green patient behavior” in ambivalent patients; [5] an increasing quantity of “green patient behavior” towards the end of the last sessions. However, our qualitative study suggests that good quality MI to improve long-term medication adherence in patients with schizophrenia, is more complex. MI-strategies comprise large parts of the MI-sessions, and in these sessions sufficient attention for both change talk and sustain talk is essential. Furthermore, “neutral talk” often serves an important role in rapport building, which is also an essential MI-component.

Legend

Therapist (top half)

dark green = question querying for change, or two-sided question

light green = reflection of change talk, or two-sided reflection

blue = MI-consistent techniques (affirm, emphasize control, permission seeking, support)

grey = other (facilitate, filler, self-disclosure, general information, raise concern, structure, advice with permission, not encodable)

yellow = question querying counter-change, of question not directed at the target behavior

orange = reflection of sustain talk, or reflection of neutral talk

red = MI-inconsistent techniques (confront, direct, warn, opinion, advice without permission)

Patient (bottom half)

green = change talk

grey = neutral talk

orange = sustain talk

Case 1. At the start of the MI-sessions this patient felt ambivalent about long-term medication use, and remained ambivalent through all sessions but tended to medication adherence.

Session 1

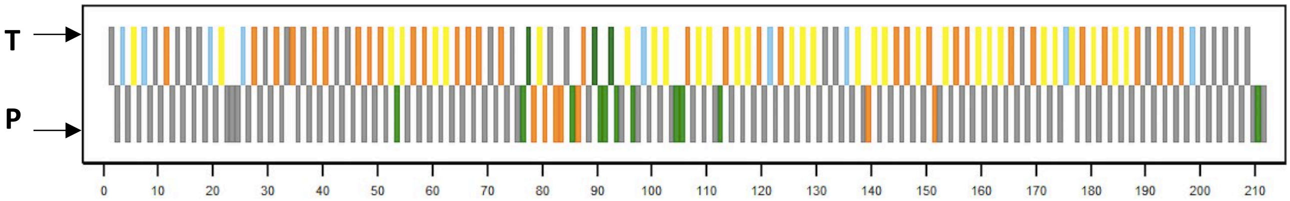

Session 2

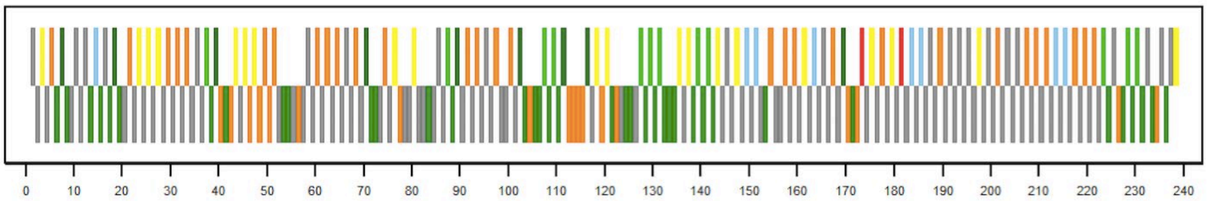

Session 3

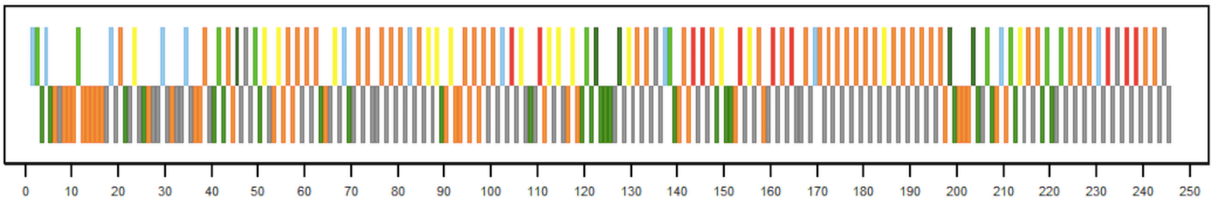

Session 4

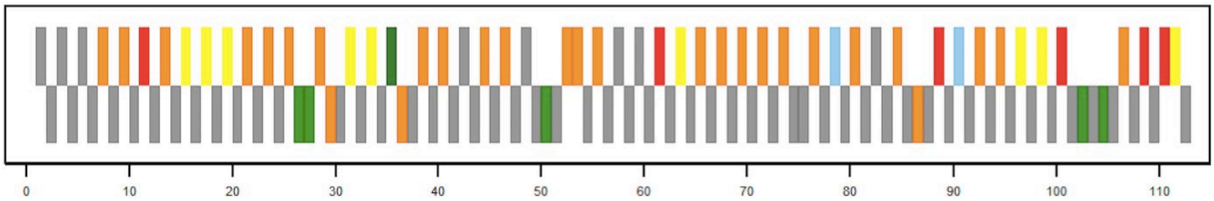

Case 2. This patient avoided discussing the topic of medication use, as visualized by the grey bars in the bottom half of the session images.

Session 1

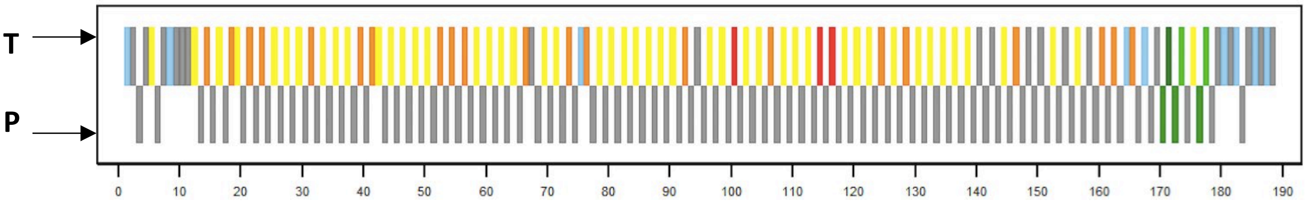

Session 2

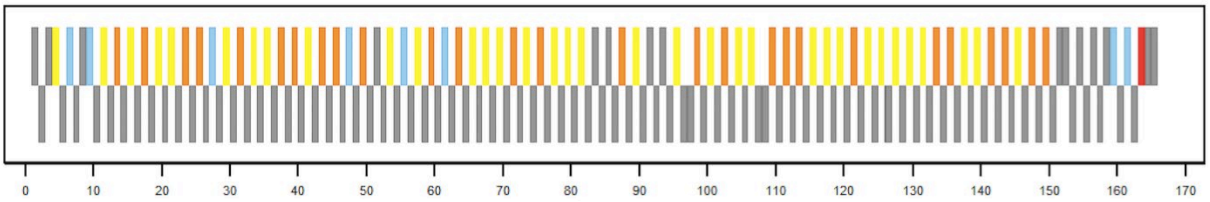

Session 3

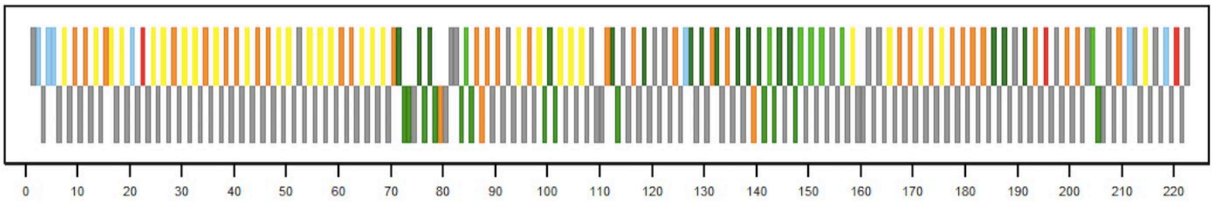

Session 4

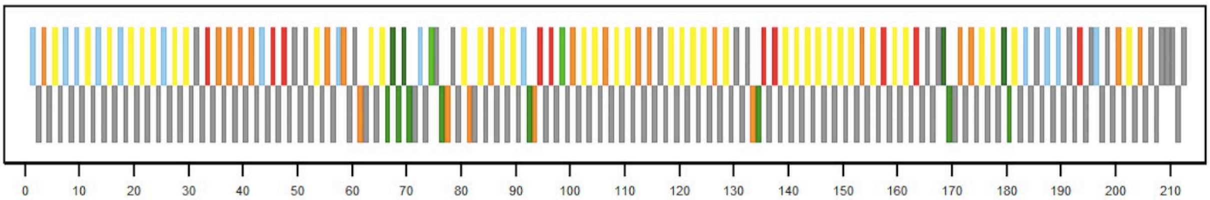

Session 5

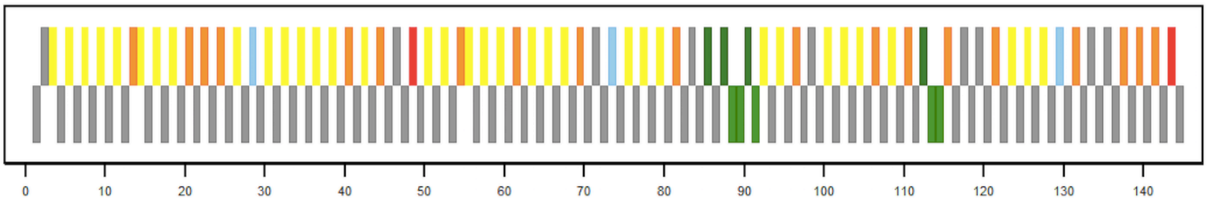

Case 3. This patient did not feel ambivalent and strongly believed in the decision to stop medication use immediately after discharge from hospital. In all sessions, the therapist and the patient explored the patient's motives and discussed the patient's perspectives. The therapist tried to influence patient's sense making and to develop discrepancy, but both attempts failed. In session 3 the atmosphere almost became unpleasant when the therapist confronted the patient four times (codes 162 – 171), but it did not become really tense, and the trusting relationship remained intact.

Session 2

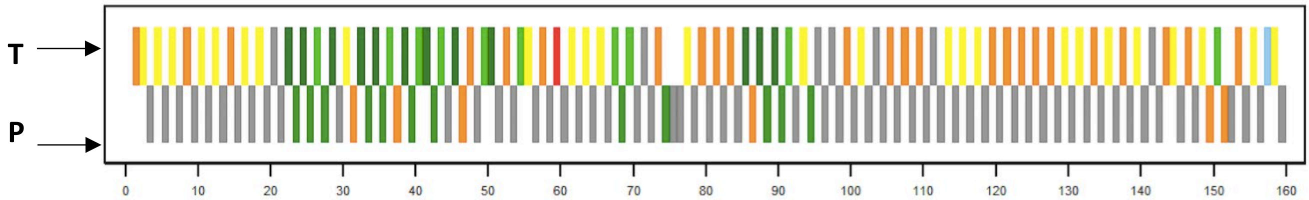

Session 3

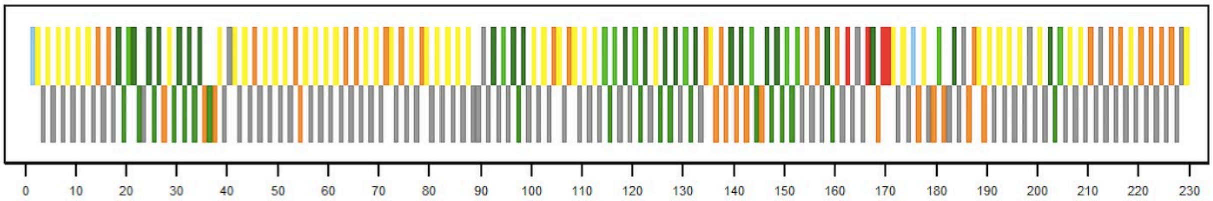

Session 4

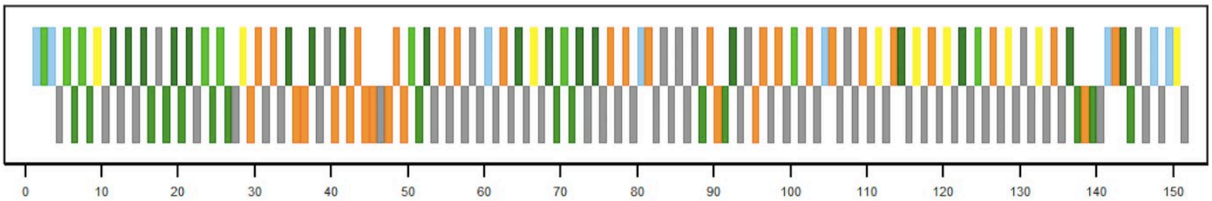

Session 5

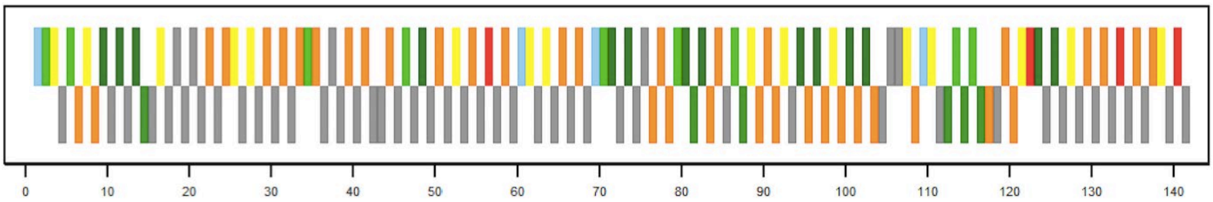

Session 6

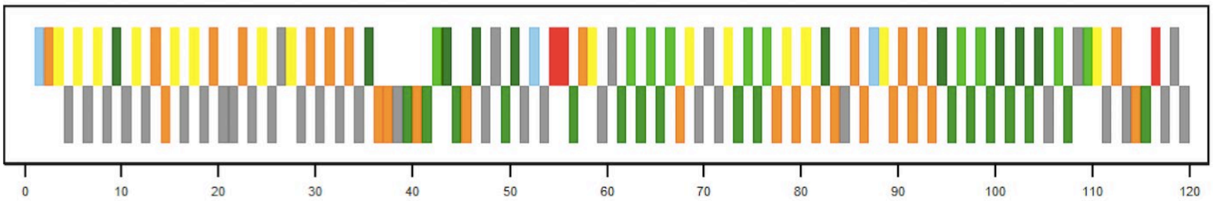

Case 4. At the start, this patient felt ambivalent about long-term medication use, and the patient remained ambivalent during all sessions. The therapist tried to persuade the patient, and expressed his opinion many times. In session 2 and session 7, the conversation was not on the target behavior. In sessions 6 and 8, the therapist and the patient performed a decision balance: exploring the pros and cons of long-term medication use.

Session 1

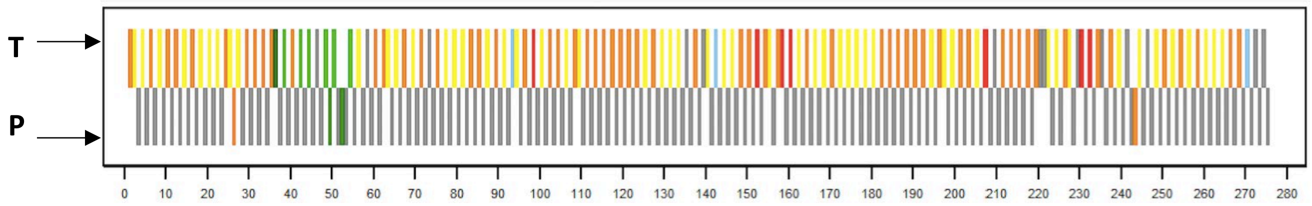

Session 2

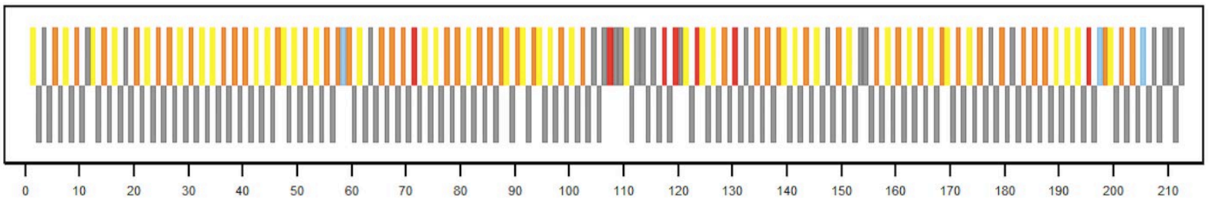

Session 3

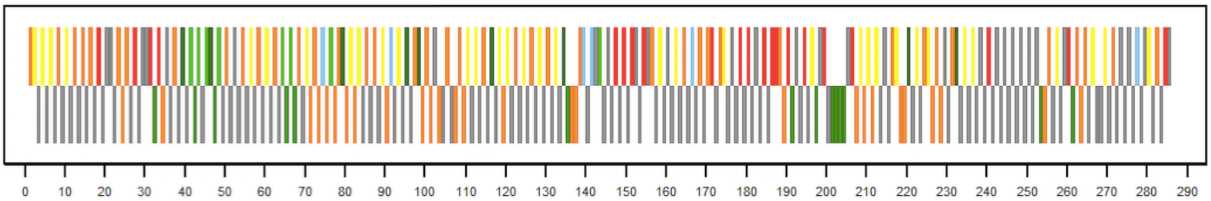

Session 4

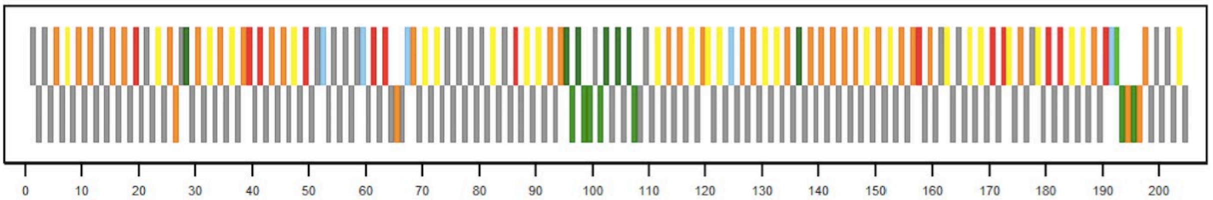

Session 5

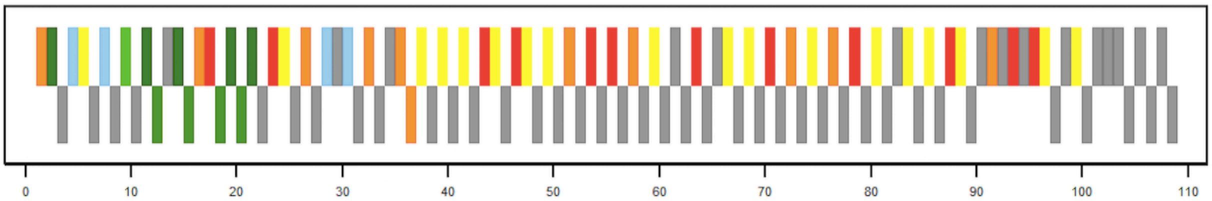

Session 6

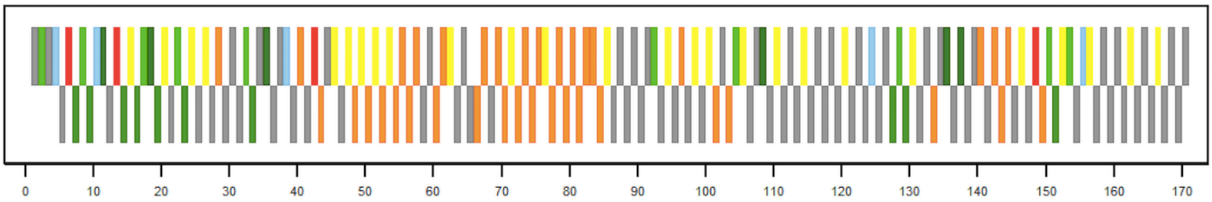

Session 7

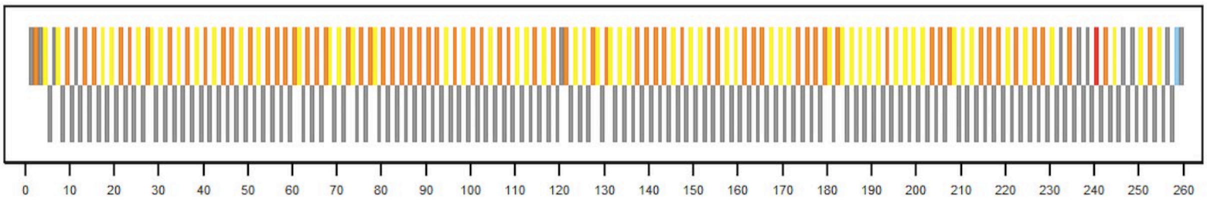

Session 8

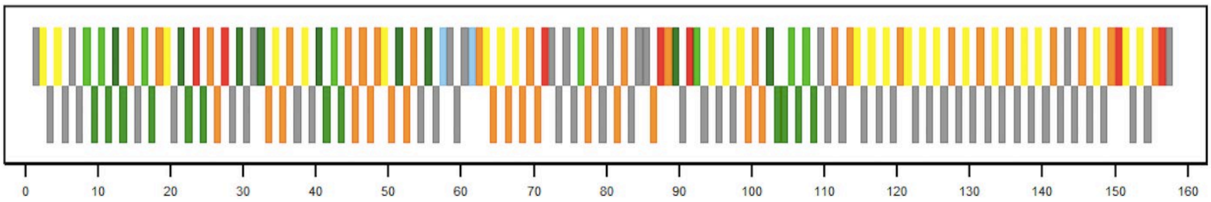

Session 9

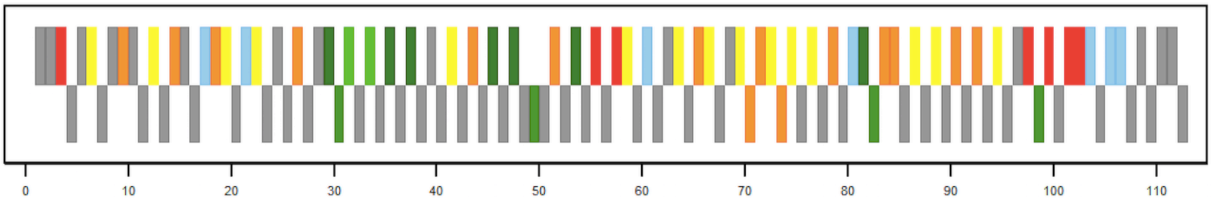

Case 5. At the start of the MI-sessions, this patient felt ambivalent about long-term medication use, which became apparent in session 2. Especially during sessions 3 and 4, the patient explored the meaning of medication in daily life. In session 5, the patient solved the ambivalence, and, in spite of the disadvantages, the patient decided in favor of long-term medication adherence.

Session 1

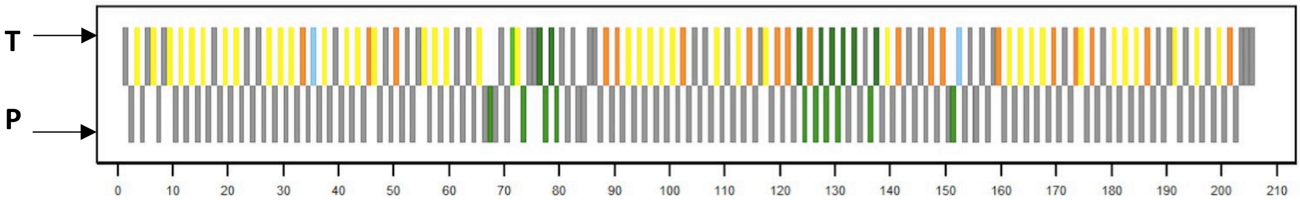

Session 2

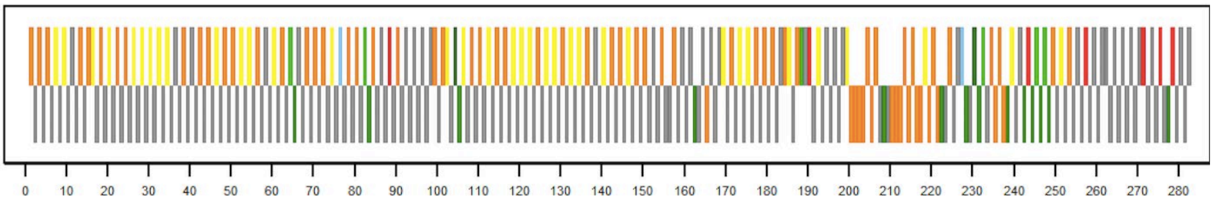

Session 3

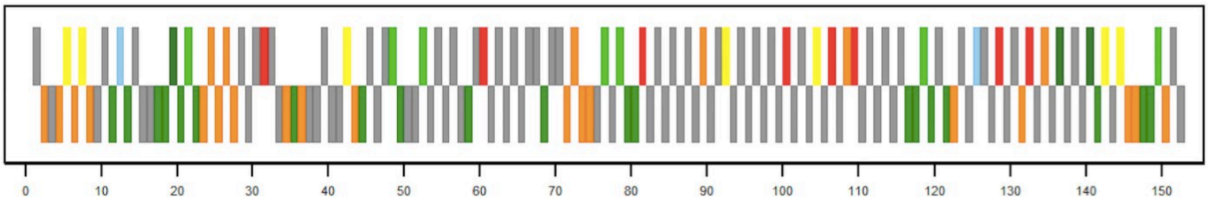

Session 4

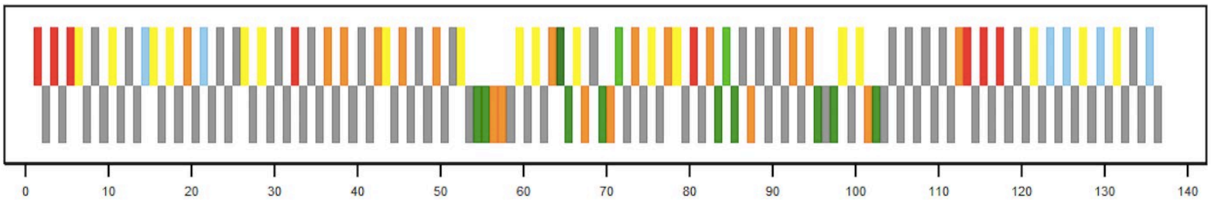

Session 5

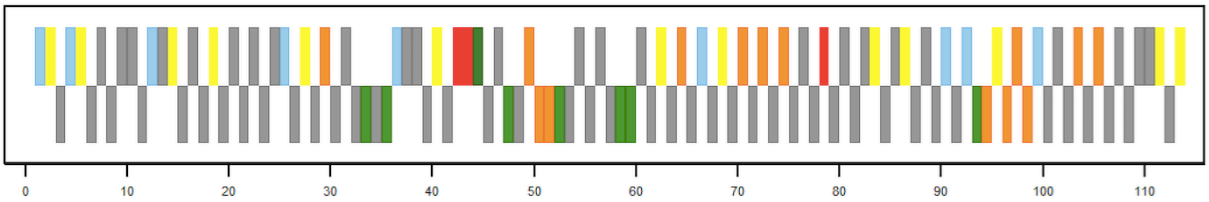

Case 6. At the start, this patient felt ambivalent about long-term medication use, and the patient remained ambivalent during all sessions. The ambivalence is dominantly present in the fourth session. Hereafter, there was a stagnation in the MI-process and the patient's ambivalence was still unchanged in the last session.

Session 1

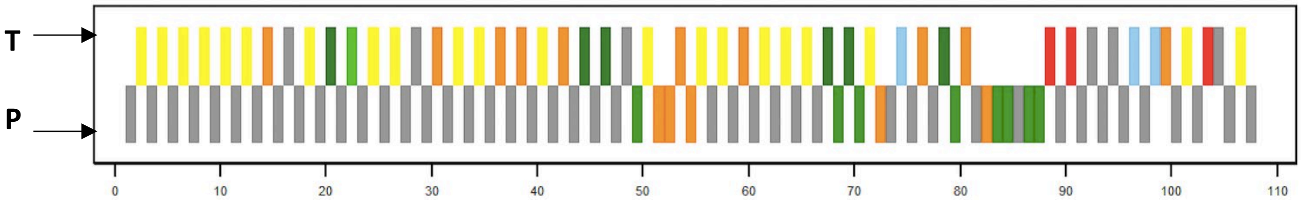

Session 2

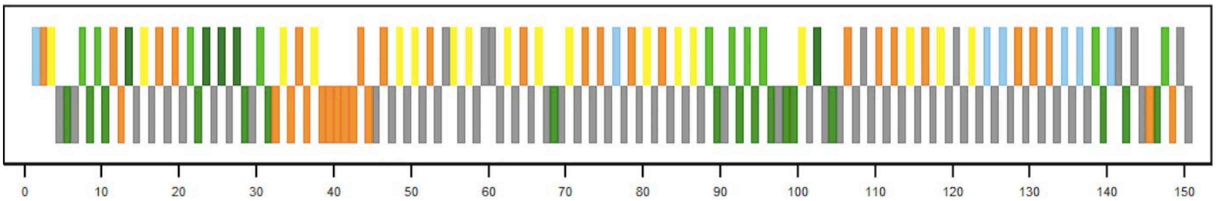

Session 3

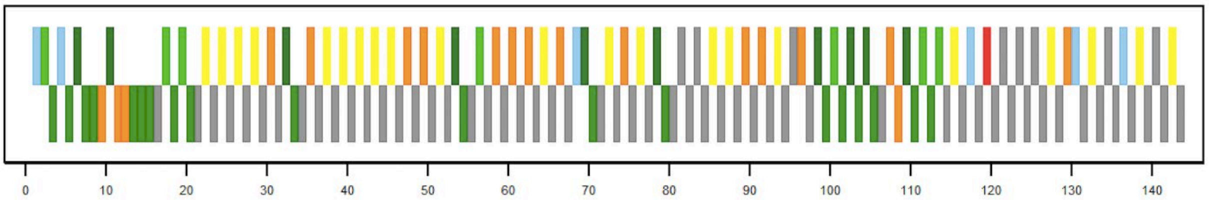

Session 4

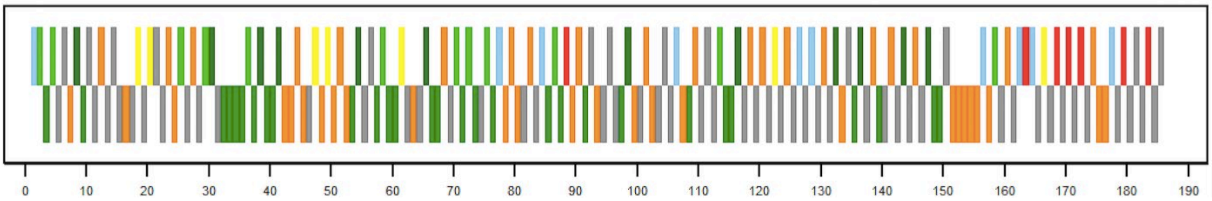

Session 5

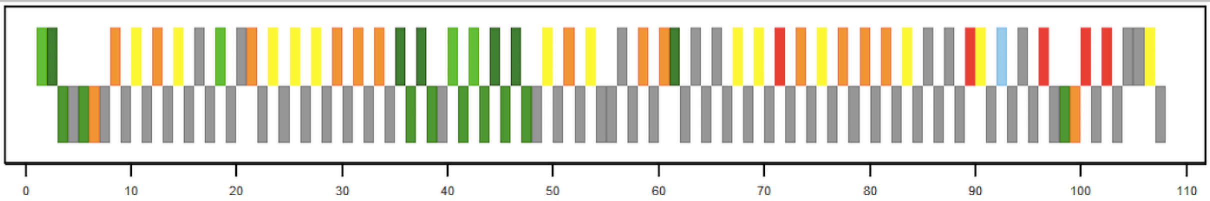

Session 6

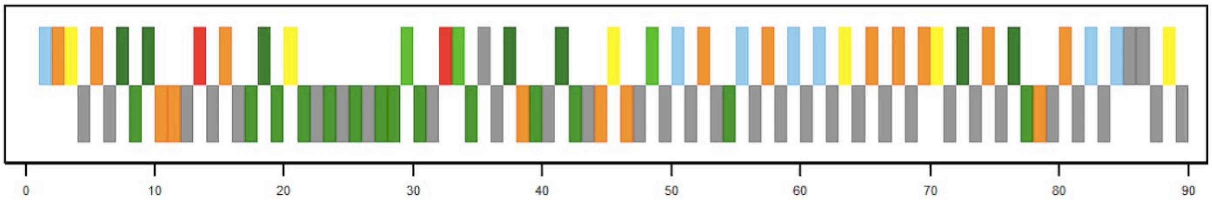

Session 7

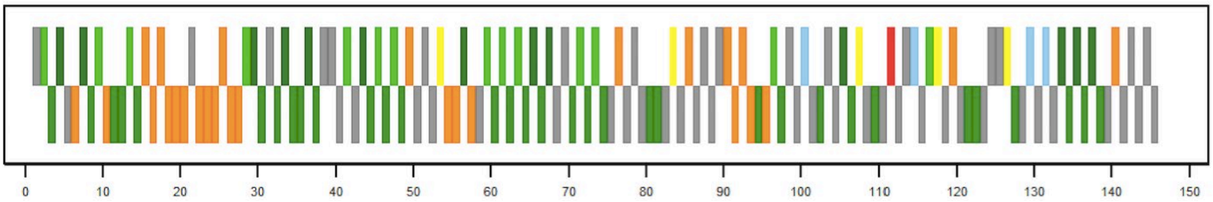

Case 7. This patient did not feel ambivalent and was convinced that medication “is of no use”. The language barrier between patient and therapist and the lack of engagement between them, led to unfruitful sessions. The change talk in session 3 and 4 was of low quality, the patient mostly followed the therapist, saying what the therapist wanted to hear.

Session 1

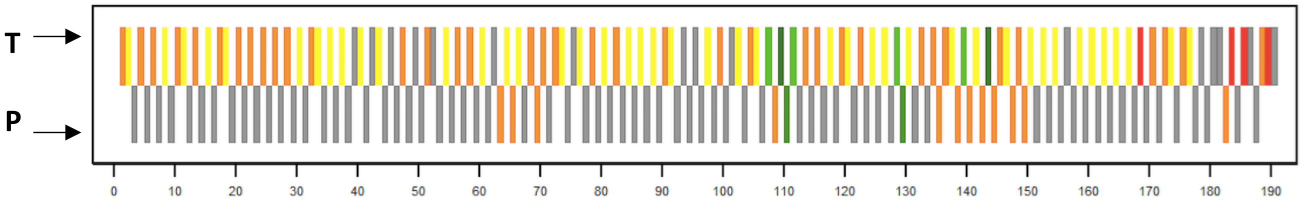

Session 2

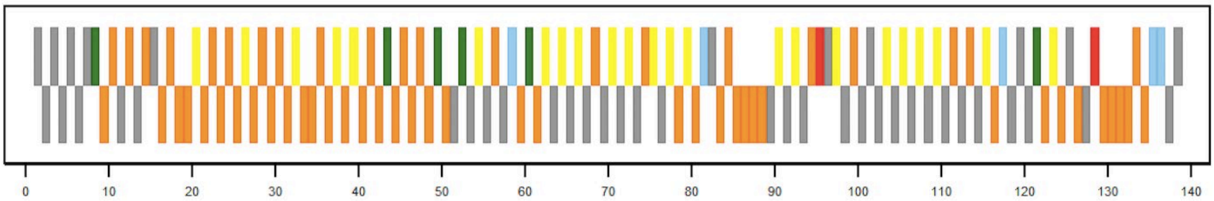

Session 3

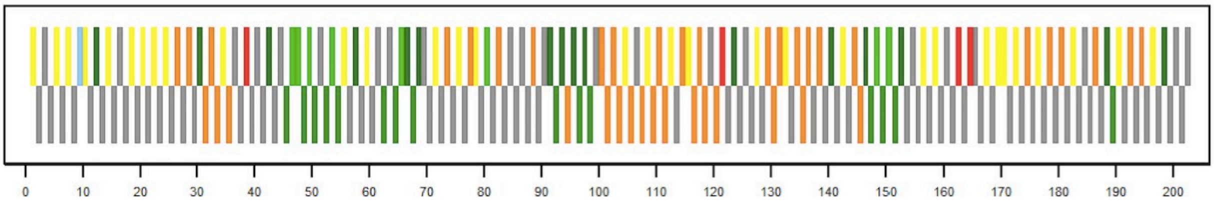

Session 4

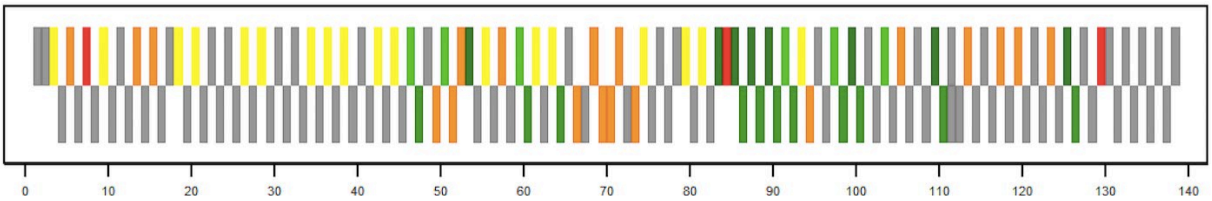

Case 8. At the start of the MI-sessions this patient felt ambivalent about long-term medication use, although in session 1, medication use was only a minor subject. To some extent, the ambivalence became apparent in the third session, but the therapist did not explore and discuss it. Particularly in the first session, there were many patient and therapist utterances. As shown by the large number of yellow bars, the high number of utterances was partly due to a large number of questions, leading to a question–answer pattern: exploring the pros and cons of long-term medication use.

Session 1

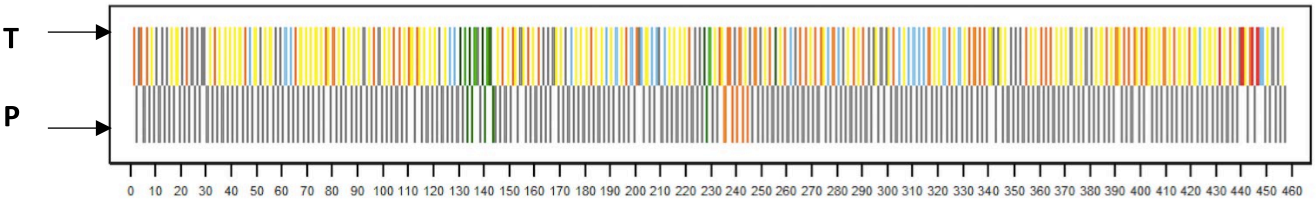

Session 2

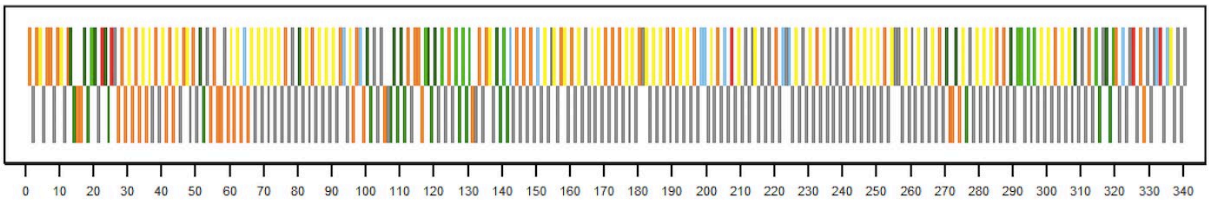

Session 3

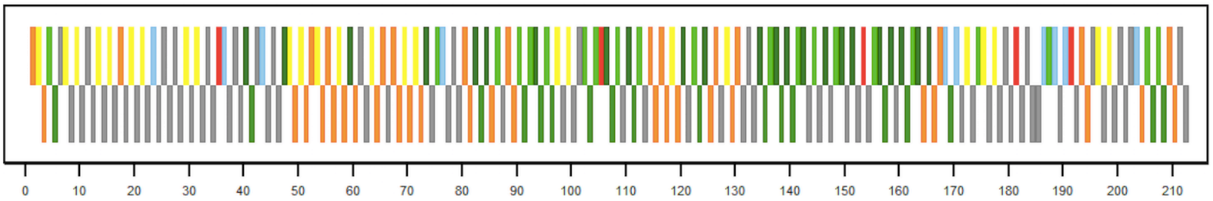

Case 9. From the start, this patient expressed motivation for long-term medication adherence. The sustain talk in session 3 was provoked by the performance of a decision balance (exploring the pros and cons of long-term medication use).

Session 1

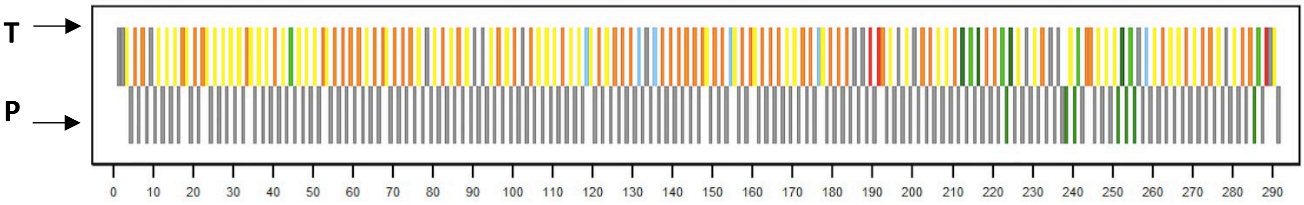

Session 2

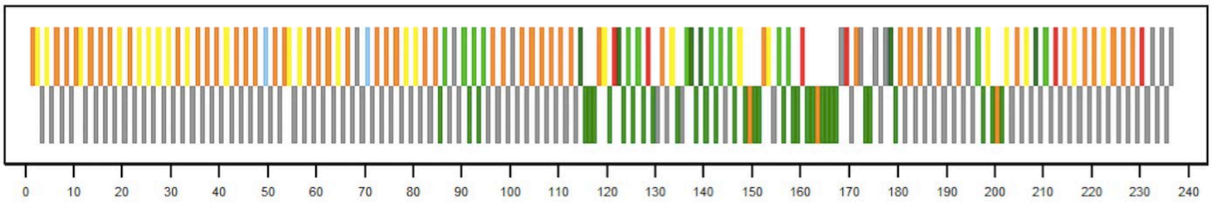

Session 3

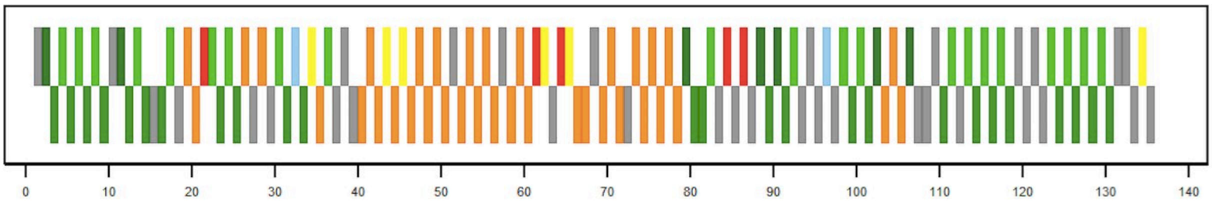

Session 4

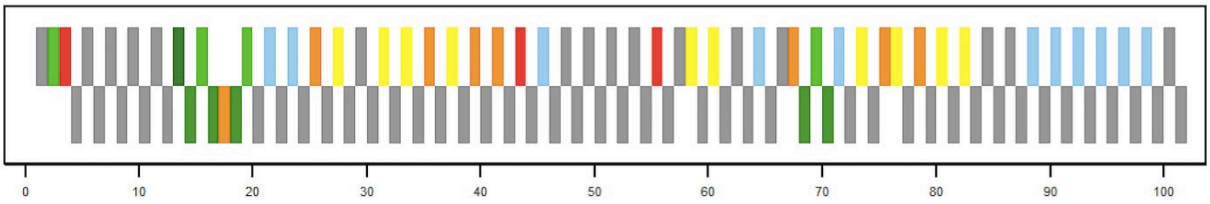

Session 5

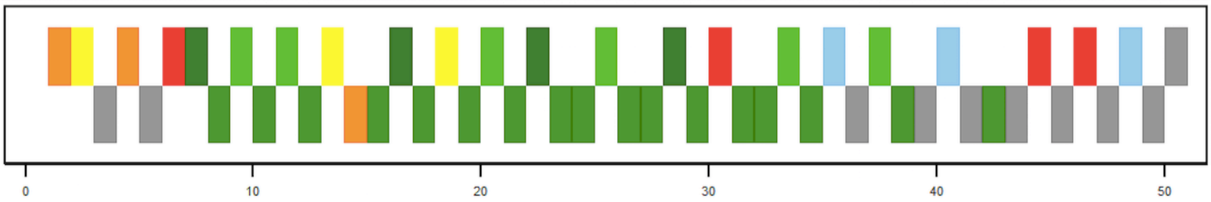

Case 10. From the start, this patient expressed motivation for long-term medication adherence. In session 2 (utterance 19-69), the therapist guided the patient to strengthen his long-term motivation for medication use.

Session 1

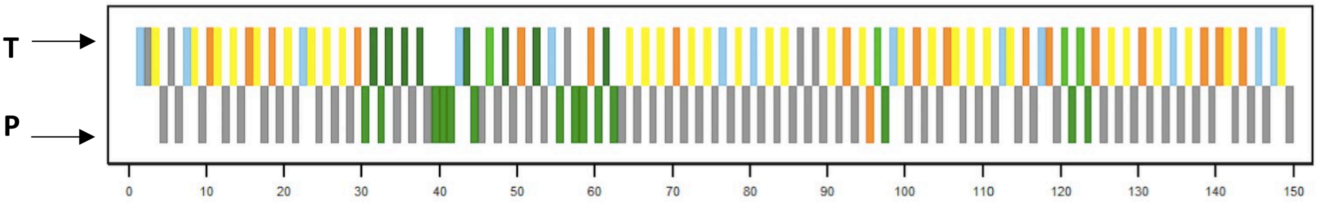

Session 2

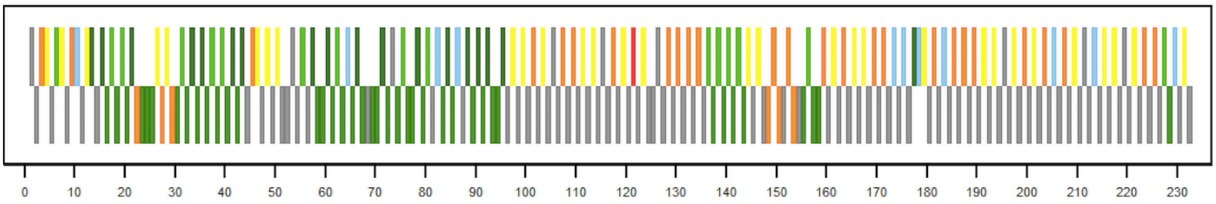

Session 3

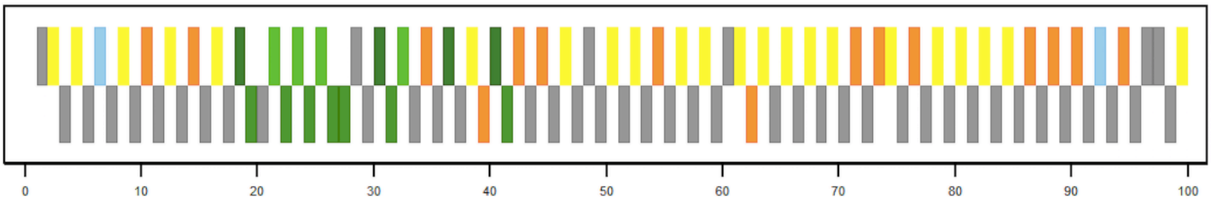

Case 11. From the start, this patient expressed motivation for long-term medication adherence. However, the level of conversation was superficial, due the patient's limited control of the Dutch language. For instance, in session 1 there were 98 patient utterances, and 51 of these consisted of only one word (mostly: "Yes."). This language barrier also led to shorter session lengths, the fifth session consisted of only 32 utterances, of which there were 15 patient utterances and of these 9 utterances of one word. In the remaining six utterances, the patient said five times that medication is important and should be taken until the psychiatrist advises otherwise.

Session 1

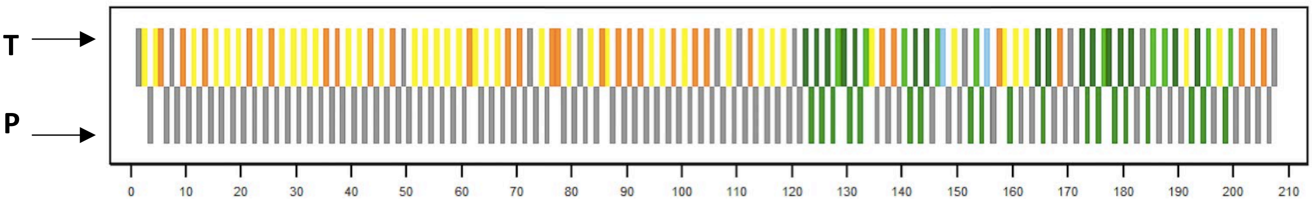

Session 2

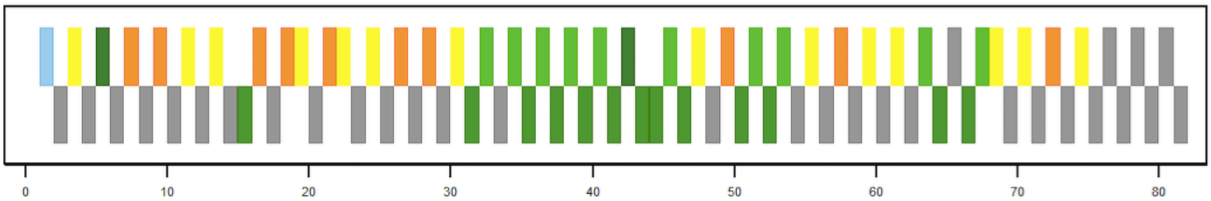

Session 3

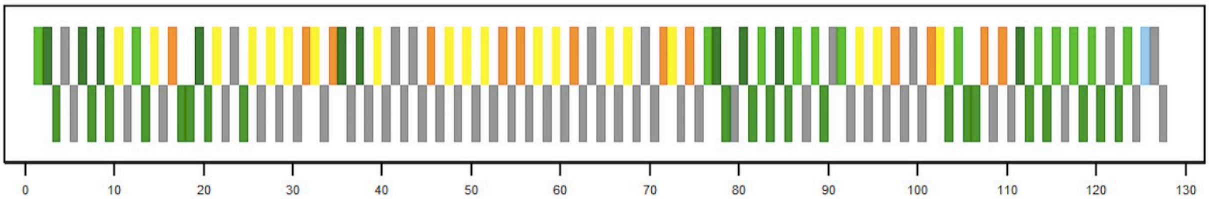

Session 4

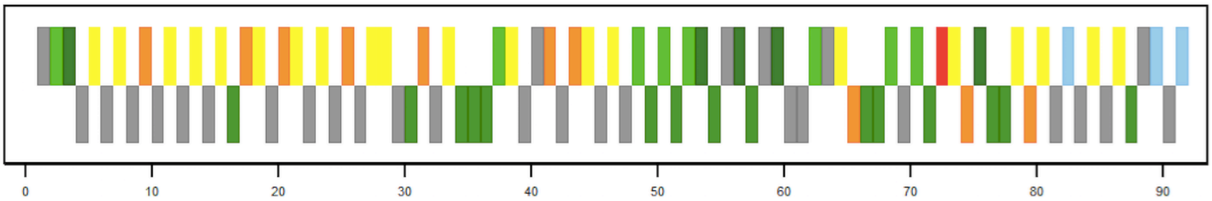

Session 5

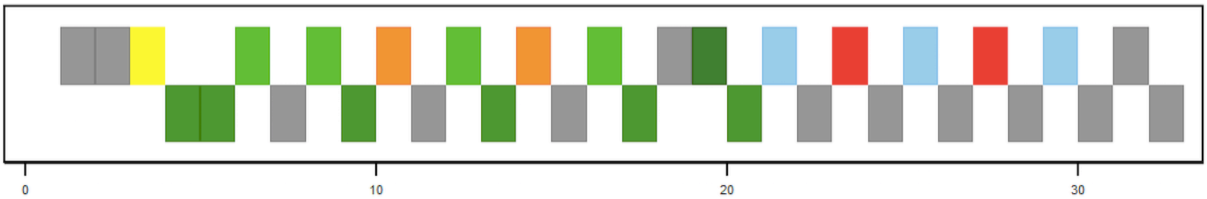

Case 12. From the start, this patient expressed motivation for long-term medication adherence. The sustain talk in the first part of session 2 was provoked by the performance of a decision balance (exploring the pros and cons of long-term medication use).

Session 1

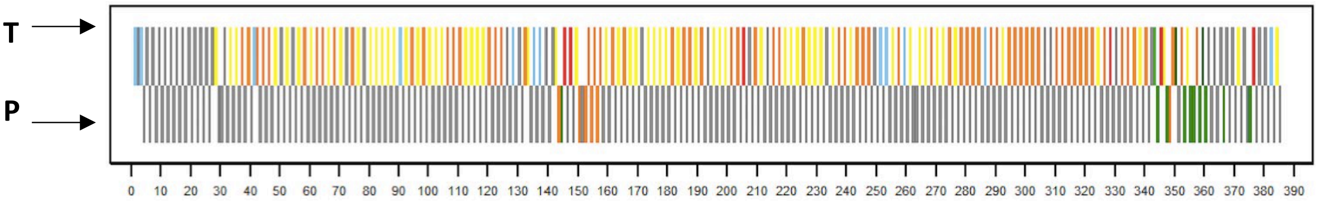

Session 2

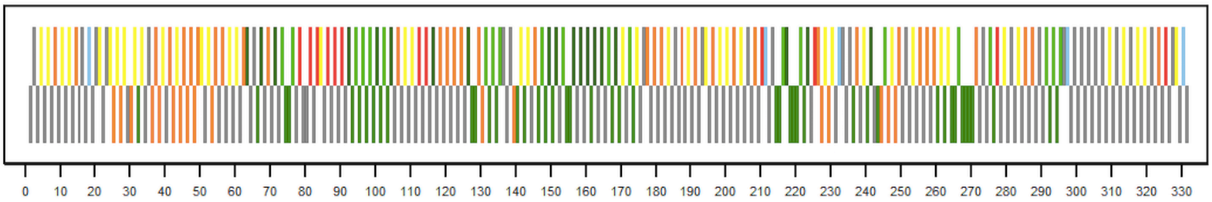

Session 3

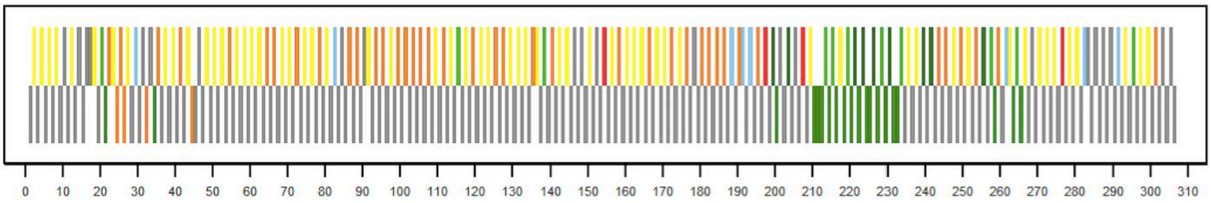

Session 4

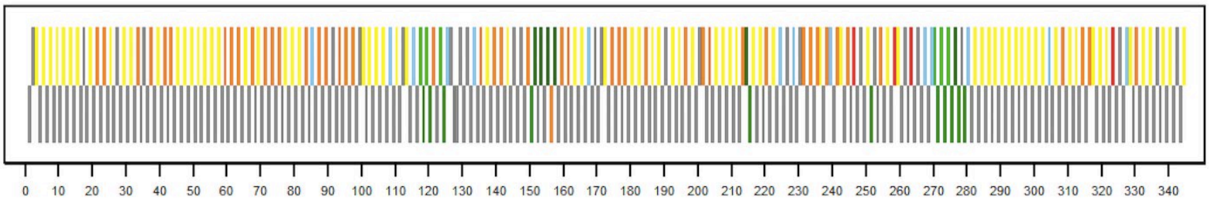

Case 13. At the start of the sessions, this patient felt ambivalent about medication use, mainly because of the side effects. In session 3, the therapist and client performed a decision balance (exploring the pros and cons of long-term medication use). During session 4, the patient switched from 'doubt/ambivalence' to the cognition 'needing medication for its effect'. This happened without an exploration of the ambivalence.

Session 1

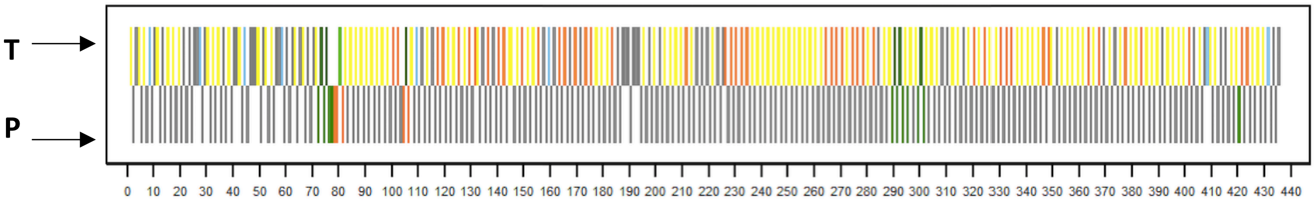

Session 2

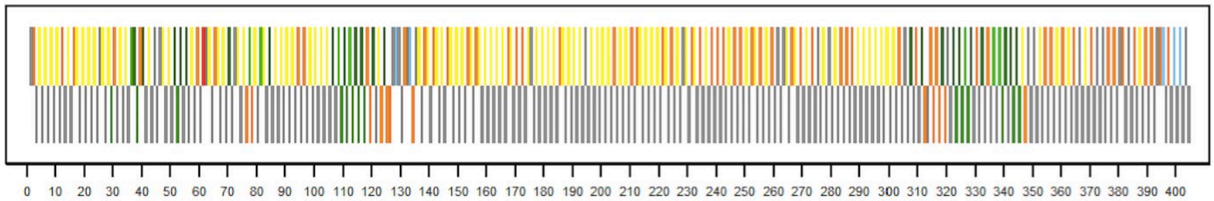

Session 3

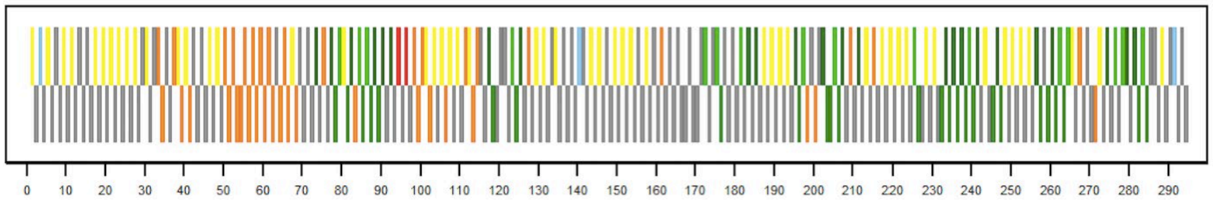

Session 4

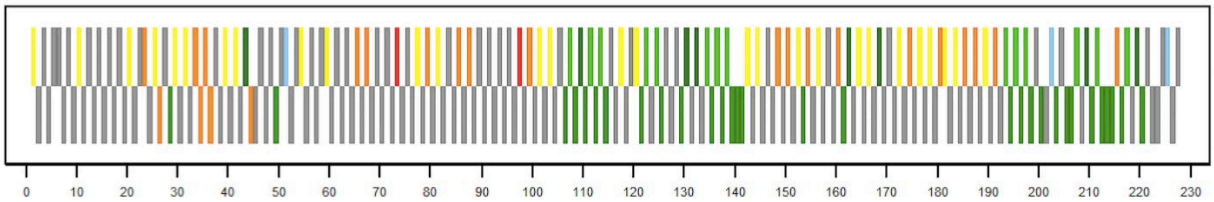

Case 14. At the start of the MI-sessions, this patient felt ambivalent about long-term medication use. In the third session, the therapist and the patient explored the patient's ambivalence. In the fourth session, the therapist and patient related important patient values to long-term adherence, after which the patient resolved the ambivalence and decided in favor of medication adherence.

Session 1

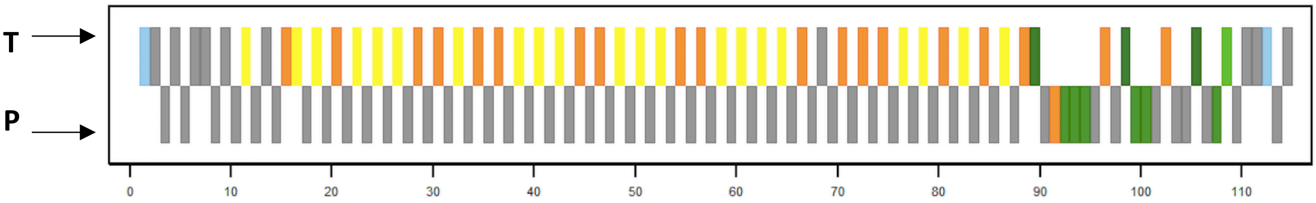

Session 3

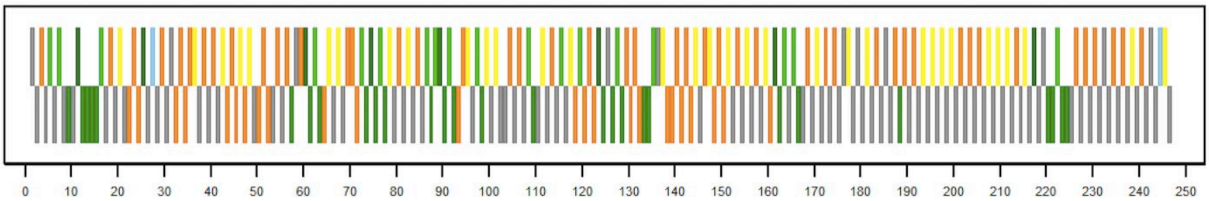

Session 4

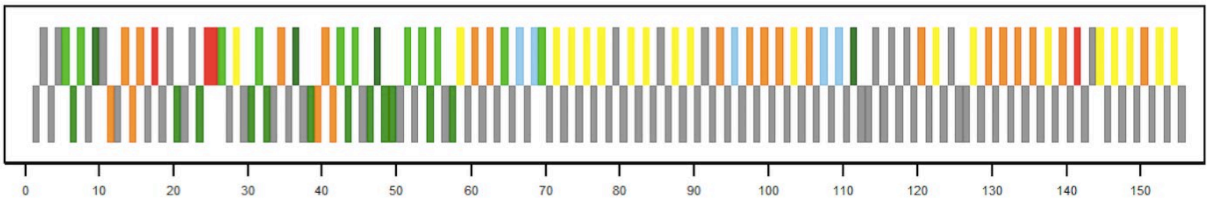

References

1. Barkhof E, Meijer CJ, Sonneville LJ, Linszen DH, De Haan L. The effect of motivational interviewing on medication adherence and hospitalization rates in non-adherent patients with multi-episode schizophrenia. Schizophrenia Bulletin 2013;39:1242-51.
2. Martin T, Moyers TB, Houck J, Christopher P, Miller WR. Motivational Interviewing Sequential Code for Observing Process Exchanges (SCOPE). Coder's manual. 2005. https://casaa.unm.edu/download/scope.pdf Accessed 28 March 2013.
3. Miller WR, Rollnick S. Motivational interviewing: helping people change. 3rd ed. New York: Guilford Press; 2013.
